# Epigenetic regulation in gynecological cancers: a paradigm shift in immunotherapy strategies

**DOI:** 10.1186/s13046-025-03509-1

**Published:** 2025-09-30

**Authors:** Chenyuan Zhao, Yang Liu, Zhuo Cui

**Affiliations:** Huludao Central Hospital, Huludao, 125000 Liaoning China

**Keywords:** Epigenetic, Gynecological cancer, Immunotherapy, TIME

## Abstract

Recent advances in immunotherapy have transformed the therapeutic landscape of gynecological cancers; however, durable responses remain limited by tumor heterogeneity and immune evasion mechanisms. Emerging evidence highlights epigenetic modifications comprising of DNA methylation, histone modifications, and RNA methylation as pivotal regulators of the tumor immune microenvironment and immunotherapy efficacy. This review comprehensively explores how these epigenetic alterations modulate immune cell infiltration, antigen presentation, immune checkpoint expression, and tumor immunogenicity across cervical, ovarian, and endometrial cancers. We also delineate the impact of specific epigenetic enzymes, such as DNMTs, HDACs, BET and RNA methyltransferases, in shaping immune responses and discuss the therapeutic potential of targeting these regulators to sensitize tumors to immune checkpoint inhibitors, cancer vaccines, cytokine based treatments and adoptive T-cell therapies. Furthermore, we examine the integration of epigenetic agents such as DNMT and HDAC inhibitors with immunotherapies in preclinical and clinical settings, emphasizing their synergistic capacity to overcome immunoresistance. By illuminating the interplay between epigenetic regulation and immune dynamics, this review underscores a paradigm shift toward precision immunoepigenetic strategies, offering a promising framework for enhancing therapeutic outcomes in gynecological malignancies.

**Graphical Abstract:**

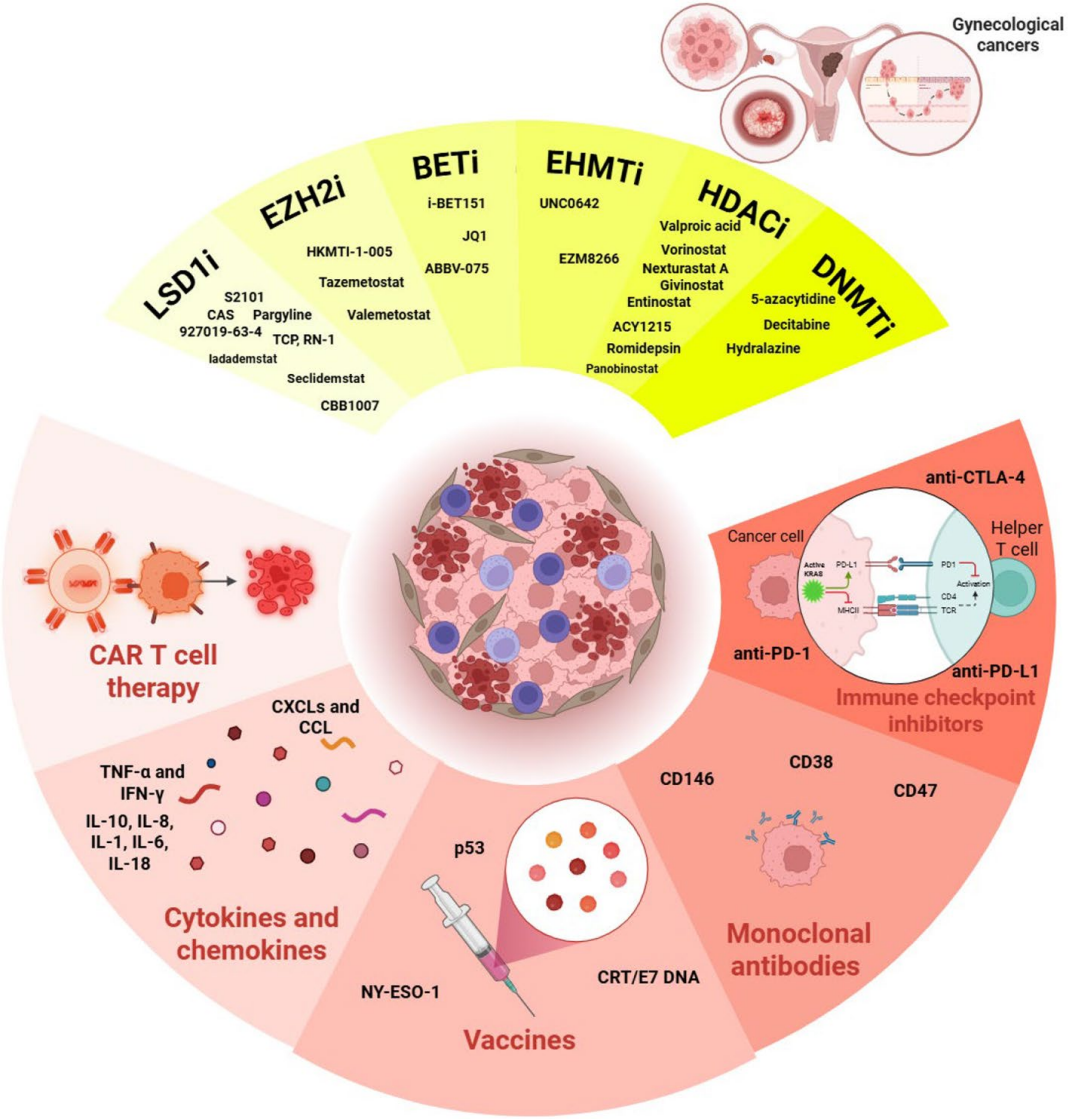

## Introduction

Gynecological cancers, ovarian, cervical, and endometrial, pose significant challenges due to their molecular complexity, aggressive behavior, and therapy resistance. These cancers drive global morbidity and mortality through genetic and epigenetic alterations that promote tumor growth and immune evasion [1]. Ovarian cancer, the deadliest of these, has a 5-year survival rate of 47%. Only 15% of cases are diagnosed early, and despite surgery and platinum-based chemotherapy, most patients relapse within three Years, with 25% showing platinum resistance at first relapse. Alternative drugs like paclitaxel or gemcitabine offer limited success [2, 3]. Similarly, cervical cancer ranks as the fourth most prevalent cancer among women And the fourth leading cause of cancer-related mortality, with approximately 604,000 new cases And 342,000 deaths reported in 2020 [4]. Linked to HPV, it involves oncogenic proteins (E6, E7, E5) that disrupt DNA And immunity. While early cases are 90% curable with surgery, chemotherapy, and radiation, advanced cases often resist treatment [5]. On the other hand, endometrial cancer, the sixth most prevalent cancer in women, had 420,242 new cases And 97,704 deaths in 2022. Its rising incidence and treatment resistance underscore the need for better understanding of its molecular drivers [6]. The rising incidence and resistance to standard treatments underscore the urgent need for a deeper understanding of the molecular and epigenetic mechanisms driving these cancers.

Epigenetic mechanisms, such as DNA methylation, histone modifications, acetylation, methylation, lactylation, and RNA methylation, regulate gene expression without altering DNA sequences, significantly impacting the tumor immune microenvironment (TIME) [7, 8]. These processes contribute to chemoresistance by silencing tumor suppressor genes, enhancing drug efflux, and disrupting DNA repair. In ovarian cancer, BRCA1 promoter hypermethylation impairs DNA repair, reducing platinum therapy efficacy, while histone deacetylation suppresses pro-apoptotic genes, decreasing chemotherapy sensitivity [9]. In cervical cancer, HPV-induced DNMT1 and HDAC upregulation leads to hypermethylation and deacetylation of apoptosis-related genes like hMLH1 and DAPK1, promoting cisplatin resistance [10]. In endometrial cancer, aberrant MLH1 methylation disrupts DNA damage response, fostering chemoresistance [11]. Epigenetic changes also influence immunotherapy outcomes by altering immune cell infiltration, antigen presentation, and checkpoint signaling, affecting treatments like immune checkpoint inhibitors (ICIs), cancer vaccines, and CAR T-cell therapies [12–14]. Notably, Cacan et al. showed that in chemoresistant ovarian cancer, DNA hypermethylation and histone deacetylation, driven by HDAC1/HDAC3 and DNMT1, suppress immune co-stimulatory molecules while upregulating immunosuppressive PD-L1 [15]. Yan et al. further demonstrated that elevated HDAC expression in gynecological cancers reduces NK and CD8 + T cell infiltration and immune-activating gene expression, an effect counteracted by HDAC inhibitors to boost anti-tumor immunity [16]. Combining epigenetic therapies, such as DNMTi and HDACi, with immunotherapy shows promise in reprogramming the TIME, enhancing tumor immunogenicity, and overcoming resistance.

Epidrugs like DNMTi and HDACi are gaining attention for their ability to reverse epigenetic silencing, upregulate tumor antigens, and enhance immune effector function, sensitizing tumors to chemotherapy and ICIs [17, 18]. For instance, DNMTi increase MHC class I and tumor antigen expression, improving CD8 + T cell recognition in ovarian and cervical cancer models [19]. HDACi restore platinum sensitivity by promoting apoptosis and activating immune pathways, such as type I interferon signaling, enhancing ICI efficacy. Preclinical and clinical studies confirm that combining epidrugs with immunotherapies increases T-cell infiltration and reduces immunosuppressive cells, overcoming immunoresistance [20, 21].

The synergistic potential of epidrugs lies in their ability to address both chemoresistance and immune suppression, offering a dual mechanism to improve therapeutic outcomes. By targeting epigenetic modifications, these therapies can upregulate immune-related genes, increase tumor-associated antigen expression, and improve immune effector function, thereby sensitizing tumors to ICIs and other immune-based approaches. Moreover, epigenetic profiling offers opportunities to identify biomarkers for patient stratification and predict treatment responses, paving the way for precision oncology. This article provides a comprehensive examination of the interplay between epigenetic regulation and immune responses in gynecological cancers. Through an in-depth review of preclinical studies, clinical trials, and emerging therapeutic combinations, we underscore the transformative potential of integrating epigenetic interventions with immunotherapy to overcome resistance, enhance anti-tumor immunity, and ultimately improve survival and quality of life for patients facing these formidable diseases and to drive a paradigm shift toward personalized, immune-centric therapeutic strategies.

## An overview of immunotherapy in gynecological cancers

Immunotherapy has transformed the treatment of gynecological cancers, including ovarian, cervical, and endometrial malignancies, by leveraging immune mechanisms to target tumors. This section reviews key immunotherapeutic strategies—cytokine therapy, cancer vaccines, ICIs, and CAR T-cell therapy—highlighting their mechanisms, efficacy, and challenges in these cancers.

### Cytokine therapy

#### Interferons (IFN)

Interferons (IFN-α and IFN-γ) exhibit cytotoxic and immunomodulatory effects in gynecological cancers. In cervical cancer, IFN-α and IFN-γ induce apoptosis in HeLa cells and long-term G1 cell cycle arrest, mediated by p57 and p27 [22]. In ovarian cancer, combining IFN-α/γ with IL-4-PE enhances cell death and survival in xenograft models [23]. A clinical trial showed IFN-γ with first-line chemotherapy extended progression-free survival in ovarian cancer from 38 to 51% at three years [24]. Locoregional IFN-α/γ-stimulated monocytes target the immunosuppressive microenvironment in resistant ovarian cancer, underscoring IFNs’ potential in personalized immunotherapy [25].

#### Interleukins

Interleukins modulate immune responses in gynecological cancers. In cervical cancer, IL-33 promotes immune evasion via IL-13 and M-MDSCs, while IL-24 inhibits proliferation and enhances apoptosis in SiHa and HeLa cells [26]. IL-12 boosts T-cell responses to HPV antigens, improving cell-mediated immunity [27]. Conversely, IL-17 drives tumor progression via JAK2/STAT3 and PI3K/Akt/NF-κB pathways [28]. In endometrial cancer, elevated IL-31 and IL-33 levels correlate with tumor stage, serving as prognostic biomarkers, while IL-11 blockade reduces tumor growth and metastasis in mouse models [29]. These findings highlight interleukins as key therapeutic targets.

### Cancer vaccines

Cancer vaccines stimulate tumor-specific immune responses in gynecological cancers. In ovarian cancer, a phase I trial of a personalized dendritic cell (DC) vaccine (OCDC) elicited robust T-cell responses, extending survival when combined with bevacizumab and cyclophosphamide [30]. In metastatic endometrial cancer, a phase I/II study of DC vaccines targeting Mucin-1 and Survivin, combined with chemotherapy, depleted suppressive myeloid cells and induced antigen-specific responses [31]. The E39 peptide vaccine targeting folate-binding protein improved disease-free survival in ovarian and endometrial cancer patients, particularly at optimal dosing [32]. Collectively, these trials illustrate that cancer vaccines, by eliciting tumor-specific immune responses, offer a safe, effective approach to improve survival and prevent recurrence in gynecological cancers. Their integration with chemotherapy and immunomodulatory agents enhances therapeutic outcomes, warranting further clinical exploration.

### Immune checkpoint inhibitors

The advent of ICIs has revolutionized cancer treatment, significantly enhancing clinical outcomes across various cancer types. The most prominent immune checkpoint molecules currently include programmed death-1 (PD-1), programmed Cell Death Ligand 1 (PD-L1), and cytotoxic T-lymphocyte–associated Antigen 4 (CTLA-4), with several well-established drugs widely employed in therapy. Additionally, emerging checkpoint inhibitors, such as B and T lymphocyte attenuators, are being explored and assessed for their potential in advancing anti-cancer immunotherapy [33].

#### PD-1 or PD-L1 Inhibitors

PD-1/PD-L1 inhibitors disrupt immunosuppressive signaling, enhancing T-cell activity in gynecological cancers. In ovarian cancer, anti-PD-L1 antibodies increased T-cell infiltration and survival in preclinical models [34]. Additionally, engineered γδ T cells secreting anti-PD-1 antibodies (Lv-PD1-γδ T cells) exhibited improved proliferation and cytotoxicity, offering prolonged survival in ovarian tumor-bearing mice without tumorigenicity risks, highlighting the versatility of PD-1/PD-L1 blockade in overcoming immunosuppressive barriers [35]. In cervical cancer, camrelizumab achieved an 80% objective response rate (ORR) in advanced cases, with PD-L1-positive tumors showing higher responses (42% ORR) [36, 37]. Similarly, balstilimab, another anti-PD-1 Antibody, yielded a 15% ORR in recurrent/metastatic cervical cancer, with a notable 20% ORR in PD-L1-positive tumors [38]. Socazolimab, an anti-PD-L1 monoclonal Antibody, further corroborated these findings, with a 15.4% ORR And a median PFS of 4.44 months in similar patient cohorts [39]. In endometrial cancer, dostarlimab And retifanlimab yielded ORRs of 42% And 51.3% in dMMR/MSI-H tumors, respectively, with tolerable safety profiles [40, 41]. PD-L1 status is a critical biomarker for ICI efficacy [42].

#### CTLA-4 inhibitors

Immune checkpoint therapies use monoclonal antibodies to enhance anti-tumor immunity by targeting pathways like CTLA-4, a T-cell receptor that inhibits activation by binding B7 proteins and boosts regulatory T-cell suppression [43]. Anti-CTLA-4 antibodies, such as ipilimumab or tremelimumab, increase T-cell activity and reduce suppression, with ipilimumab yielding lasting responses in ~ 20% of melanoma patients, though response rates in other solid tumors are < 10%. In ovarian cancer, the tumor microenvironment promotes immune suppression via CTLA-4, impairing tumor-infiltrating lymphocytes (TILs) [44]. Adding CTLA-4-blocking antibodies during TIL culture from ovarian tumors enhances CD8 + T-cell expansion and anti-tumor potency, improving TIL-based adoptive cell therapy outcomes. However, anti-CTLA-4 therapy’s potential in gynecological cancers is underexplored, and its modest monotherapy efficacy suggests combination strategies may better overcome immunosuppressive barriers [45].

#### Combining PD-1 or PD-L1 Inhibitors and CTLA-4 Inhibitors

Immunotherapy, particularly PD-1/PD-L1 and CTLA-4 checkpoint inhibitors, has transformed gynecological cancer treatment, showing efficacy in uterine, ovarian, and endometrial cancers [46, 47]. This overview highlights their effectiveness, safety, and mechanisms, focusing on PD-1/PD-L1 blockade.

In recurrent, metastatic, or persistent cervical cancer (R/M CC), combining PD-1/PD-L1 and CTLA-4 inhibitors addresses limited treatment options. A phase II trial of balstilimab (anti-PD-1) and zalifrelimab (anti-CTLA-4) in 155 women with R/M CC achieved a 25.6% ORR, with 10 complete responses And 22 partial responses, higher in PD-L1-positive tumors [48, 49]. Cadonilimab, a bispecific PD-1/CTLA-4 Antibody, with standard therapies in 82 patients, yielded a 70.4% ORR (28.4% CR) And 76.5% disease control rate, with partial responses in two of six patients with prior immunotherapy exposure. Grade ≥ 3 immune-related adverse events occurred in 18.3%, mainly hypothyroidism and rash, with no treatment-related deaths [50]. Ovarian cancer, highly lethal, benefits from PD-1/PD-L1 and CTLA-4 inhibitors targeting the immunosuppressive microenvironment. In a preclinical rat model, OncoTherad with erythropoietin (EPO) reduced angiogenesis, decreased VEGF, increased endostatin, and downregulated PD-1/PD-L1 and CTLA-4, altering immunosuppressive cells and histopathology [51]. A phase II trial of ipilimumab (anti-CTLA-4) and nivolumab (anti-PD-1) in 17 patients with rare non-epithelial ovarian cancers reported a 25% ORR in granulosa cell tumors, with one CR and one PR lasting over four Years, And a 50% clinical benefit rate [52].

In endometrial cancer, PD-1/PD-L1 and CTLA-4 inhibitors tackle complex immunotherapy responses, especially in MMR-proficient, PD-L1-negative tumors. A stage IV case showed a durable partial response to nivolumab And ipilimumab beyond 12 months [53]. In a BLT humanized mouse model, combining anti-PD-L1, anti-CTLA-4, and anti-IFN-β reduced tumor growth by 96.2%, surpassing PD-L1/CTLA-4 inhibition alone (82.9%), by decreasing T-cell exhaustion markers [54]. PD-1/PD-L1 and CTLA-4 inhibitors enhance antitumor immunity across gynecological cancers, with high ORRs in PD-L1-positive cervical tumors, microenvironment modulation in ovarian cancer, and novel combinations overcoming resistance in endometrial cancer. Personalized PD-1/PD-L1 blockade strategies are key to optimizing outcomes.

### Chimeric antigen receptor-T cell therapy

Adoptive cell therapy (ACT) utilizes tumor-reactive immune cells to target cancer cells with specific surface markers. It includes allogeneic and transgenic T-cell therapies, such as CAR-T and TCR therapies, where T cells are genetically modified to express CARs or T-cell receptors (TCRs) to enhance tumor targeting [55].

CAR-T cell therapy, a key immunotherapeutic approach, modifies T cells to target tumor-associated antigens, showing promise for gynecological cancers. T cells are extracted, engineered with a CAR (antigen-binding domain, hinge, transmembrane domain, and signaling domains like CD28, 4-1BB, CD3ζ), and reinfused to attack cancer cells expressing antigens like MUC16, FOLR1, or EpCAM. This triggers cytotoxicity and cytokine release, though challenges like tumor heterogeneity and immunosuppression persist [56, 57]. In ovarian cancer, MUC16-targeted MSLN-CAR T cells showed strong cytotoxicity against MUC16-positive OVCAR3 cells in vitro and reduced tumor volumes in mouse models, with peritoneal delivery achieving sustained remission [25, 26]. EpCAM-CAR T cells effectively targeted SKOV3 cells, reducing tumor size in xenografts [27]. nfP2X7-CAR T cells selectively killed OVCAR3 and OVCAR5 cells while sparing normal cells, reducing tumor Burden in mice for 7–8 weeks [28]. Dual-target CAR-T cells (e.g., FOLR1/MSLN or PD1-antiMUC16) outperformed single-target therapies, enhancing proliferation, cytokine secretion, and tumor lysis, with improved survival in mouse models [29]. For cervical cancer, NKG2D CAR-T cells lysed NKG2DL-positive cells, suppressed tumor growth in vivo, and showed no off-target toxicity [31]. TCR mimic nanobody-based CAR-T cells targeting HPV16 E6/E7 peptides killed CaSki and SS4050 cells and inhibited tumor growth in xenografts [32]. These findings collectively position CAR-T cell therapy as a transformative, targeted immunotherapeutic strategy, with ongoing research ready to refine its efficacy and pave the way for clinical translation in gynecological cancers.

## Epigenetic modification-mediated regulation of immune components, TIME, and immunotherapy in gynecological cancers

### DNA methylation

DNA methylation is a pivotal epigenetic mechanism that significantly influences gene expression in gynecological cancers. This process involves the addition of a methyl group to the 5′ position of cytosine within CpG islands, forming 5-methylcytosine (5mC), a modification primarily catalyzed by DNA methyltransferases (DNMTs), which act as the writers, of methylation marks [58]. Among the key enzymes, DNMT3a and DNMT3b facilitate de novo methylation, establishing new methylation patterns, while DNMT1 maintains these patterns during cell division [59]. Conversely, DNA demethylation is mediated by the Ten-eleven translocation (TET) enzyme family, TET1, TET2, and TET3, which serve as erasers, by oxidizing 5mC into 5-hydroxymethylcytosine (5hmC) and further derivatives [60]. Complementing these activities, a class of proteins known as methyl-CpG-binding domain proteins (MBDs), including MBD1, MBD2, MBD3, MBD4, and MeCP2, function as readers, of DNA methylation. These proteins specifically recognize and bind to methylated DNA, recruiting chromatin remodeling complexes, histone deacetylases, and other epigenetic regulators to silence gene transcription. In the context of gynecological cancers, aberrant activity of MBDs reinforces the transcriptional repression of tumor suppressor and immune-related genes, contributing to immune evasion and therapeutic resistance. This tripartite classification of DNA methylation machinery—DNMT3a and DNMT3b as de novo writers, DNMT1 as a maintenance writer, and MBDs as readers—highlights the intricate regulatory network modulating the TIME and shaping responses to immunotherapy [61]. This dynamic interplay between methylation and demethylation orchestrates gene regulation, shaping the TIME and influencing the efficacy of immunotherapy in gynecological cancers. In gynecological cancers, DNA methylation significantly modulates immune responses by regulating immune-related genes, antigen presentation, and immune cell infiltration**,** offering potential biomarkers and therapeutic targets [62, 63]. Specifically, hypermethylation often suppresses immune-activating genes, while hypomethylation can enhance immunosuppressive pathways, affecting the efficacy of immunotherapies such as ICIs [64]. This subsection explores the intricate role of DNA methylation in cervical, ovarian, and endometrial cancers, highlighting its influence on TIME and its critical implications for immunotherapy**.**

In cervical cancer, DNA methylation profoundly regulates immune components and immunotherapy responses by altering the expression of key immune genes within the TIME, contributing to immune evasion and tumor progression [65]. Hypermethylation of the IFN-γ gene promoter is significantly higher in cervical cancer tissues compared to normal or cervical intraepithelial neoplasia (CIN) tissues, leading to reduced IFN-γ expression. This suppression impairs Th1-mediated cellular immune responses critical for combating HPV infection, thereby promoting viral persistence and cervical tumorigenesis [66]. Similarly, hypermethylation of antigen presentation machinery (APM) genes, including TAP1, TAP2, LMP7, tapasin, and ERp57, correlates with downregulation the human leukocyte antigen (HLA)-I expression, diminishing CD8 + T cell recognition and enabling tumor cells to escape immune surveillance. This downregulation is particularly pronounced in HPV16-positive cases, where methylation of these gene promoters is linked to viral oncogene activity, suggesting an epigenetic strategy for immune evasion. Conversely, hypomethylation of HAVCR2 and LGALS9 promoters, encoding the immunosuppressive molecules Tim-3 and galectin-9, increases their expression in cervical cancer cells, fostering an immunosuppressive TIME and correlating with poorer overall survival [67]. The IL-10 gene promoter exhibits lineage-specific methylation in cervical cancer cell lines, silencing IL-10 production in neoplastic cells, while stromal cells or infiltrating lymphocytes produce IL-10, contributing to local immune suppression [68]. A nine-gene prognostic signature, incorporating methylation-related genes such as CCR7, CD6, POU2AF1, DNASE1L3, and IL12RB2, has been developed to predict survival and immunotherapy response. This signature highlights the role of methylation in immune cell trafficking (e.g., CCR7 in effector cell migration) and lymphocyte development (e.g., CD6 and POU2AF1), with high-risk patients showing lower immune scores, reduced CD4 + and CD8 + T cell infiltration and downregulated HLA family genes for instance HLA-A, HLA-B and immune checkpoints. Gene set enrichment analysis further reveals that high-risk groups exhibit enriched proliferation pathways, such as Myc and E2F targets, while low-risk groups show enhanced inflammatory and apoptotic pathways, underscoring methylation’s role in shaping TIME dynamics [69]. Therapeutically, demethylating agents Like 5-Aza-CdR reverse methylation patterns, upregulating Tim-3/galectin-9 expression and potentially enhancing ICI efficacy by restoring immune signaling. Additionally, drugs such as lenvatinib, a multikinase inhibitor; panobinostat, an histone deacetylases (HDAC) inhibitor; and mTOR inhibitors target methylation-driven pathways, showing synergistic effects with immunotherapy by inhibiting tumor-promoting pathways like angiogenesis and cell proliferation. The interplay between HPV oncoproteins and methylation is notable, as E6/E7 upregulate enhancer of zeste homolog 2 (EZH2) And Histone 3 Lys 27 trimethylation (H3K27me3), which recruit DNMT3A to methylate immune gene promoters, further suppressing immune responses. These findings emphasize the pivotal role of methylation-targeted therapies in overcoming immunosuppression and enhancing immunotherapy outcomes in cervical cancer, with ongoing research needed to validate these approaches in clinical settings [70].

Ovarian cancer exhibits distinct methylation patterns that shape the TIME and influence immunotherapy outcomes**.** Research identifies differentially methylated sites (DMSs) in IRGs, clustering ovarian cancers into hypermethylated (cluster 1) and hypomethylated (cluster 2) subtypes. Cluster 2, characterized by hypomethylation of genes like RORC, S100A13, and TNF, correlates with higher tumor mutation burden (TMB) and better immunotherapy responses, as higher TMB generates neoantigens that activate immune responses [71]. Hypomethylating agents (HMAs) like guadecitabine, when combined with anti-PD-1 agents like pembrolizumab, enhance immune signaling by upregulating interferon pathways and transposable elements, sensitizing tumors to ICIs**,** although clinical trials show modest responses [72]. Correlative analyses reveal that patients with durable clinical benefit have increased CD8 + T cell and B cell proximity to tumor cells, alongside tertiary lymphoid structures, suggesting methylation-driven immune cell distribution as a predictor of ICI success. However, suboptimal HMA dosing and tumor penetration challenges highlight the need for optimized epigenetic therapies to fully harness methylation’s role in immunotherapy [73]**.** In endometrial cancer, DNA methylation regulates immune infiltration and immunotherapy potential through genes like VTCN1 (B7-H4). Hypomethylation of the VTCN1 promoter leads to its overexpression, inhibiting CD8 + T cell infiltration and promoting tumor progression, which is associated with poor prognosis. Conversely, hypermethylation of PGR and GYPC correlates with reduced expression and worse outcomes, underscoring methylation’s dual role in immune regulation. The interplay between methylation and immune cell distribution, analyzed via algorithms like CIBERSORT, reveals VTCN1-driven immunosuppression as a key mechanism in endometrial cancer development [74]. DNMT inhibitors like azacitidine and decitabine have shown potential to reverse these effects by reactivating silenced immune genes, enhancing T-cell infiltration, and improving immunotherapy efficacy. However, the application of hypomethylating agents (HMAs) is not without concern. While these agents restore tumor suppressor and immune-related gene expression, they may also inadvertently activate oncogenes. A notable example is *SALL4*, a known oncogene in hematologic malignancies, which has been observed to become upregulated following HMA treatment in a subset of patients with myelodysplastic syndrome (MDS). This upregulation, driven by locus-specific demethylation, correlates with poorer outcomes and disease progression. Interestingly, only a fraction of patients exhibited this effect, suggesting that DNA demethylation may require additional chromatin remodeling events, such as enhancer–promoter interactions, to maintain oncogene expression. These findings emphasize the dual-edged nature of HMAs and the importance of monitoring unintended oncogenic activation during epigenetic therapy. In gynecological cancers, where similar mechanisms could plausibly occur, integrating biomarkers like *SALL4* could improve the safety and precision of future epigenetic-immunotherapy approaches [75]. Thus, while HMAs hold significant therapeutic promise in reprogramming the TIME, a balanced understanding of both their immunostimulatory and oncogenic potential is essential for clinical translation. This evolving understanding of methylation dynamics highlights the need for personalized strategies that weigh therapeutic benefits against possible risks of oncogene reactivation.

Targeting methylation patterns with demethylating agents could restore immune activation, potentially enhancing ICI efficacy, offering a promising avenue for personalized therapies in endometrial cancer. Linking these cancers, DNA methylation emerges as a common thread modulating the TIME across gynecological malignancies. In cervical cancer, hypermethylation of immune-activating genes like IFN-γ and APM components fosters immune evasion, while in ovarian cancer, hypomethylation enhances immunogenicity, impacting ICI responses. Endometrial cancer highlights hypomethylation-driven immunosuppression via VTCN1, underscoring the need for tailored epigenetic interventions (Table 1). Across all three cancers, methylation-targeted therapies, particularly HMAs, hold promise for reversing immunosuppressive TIME states, enhancing immunotherapy efficacy**,** and paving the way for precision medicine in gynecological oncology. Future research should focus on optimizing HMA delivery and integrating methylation signatures with immunotherapeutic strategies to improve clinical outcomes.

**Table 1:** Epigenetic modifications and their Immune-related effects in gynecologic cancers

Methylation						
**Epigenetic modification**	**Targeted by epigenetic modification**	**Downstream immune components**	**Cancer type and cell lines**	**Study type**	**Highlights**	**Ref**
↑Methylation	BRCA1	PD-L1	Ovarian	Observational cross-sectional analytical study	“BRCA1 hypermethylation linked to serous ovarian cancer, no correlation with PD-L1 expression.”	[88]
↑Methylation		↓CD8 + tumor-infiltrating lymphocytes (TILs), ↓TAP1	high-grade serous carcinoma (HGSC)	Retrospective cohort study with molecular and clinical e analyses	“Higher methylation impairs antigen presentation, promoting immune evasion.”	[89]
↓Methylation		↑KP-OVA-52	Ovarian SK-OV-3 And SNU 840	In vitro	“Demethylation induces KP-OVA-52, a potential immunotherapy target.”	[90]
↑Methylation	Protein arginine methyltransferase 5 (PRMT5)	IFN-γ, ↓TNF-α and ↓granzyme B in T cells JAK/↑STAT1/↑PD-L1 ↑TIM-3	Cervical Siha, Ms751, HeLa, and Hela229	In vitro and in vivo	“PRMT5 knockdown enhances T-cell response, regulates PD-L1 via STAT1.”	[11]
↑Methylation	Erb-B2 Receptor Tyrosine Kinase 3 (ERBB3)	↑MDSC, M1 to M2 polaryzation, ↓IFN-γ ↑TIGIT, ↑CTLA4, ↑PD-L1 CXCL9, CXCR5	CESC AV3, Hela	Retrospective observational with bioinformatics analysis	“ERBB3 methylation correlates with immunosuppressive TIME.”	[91]
Methylation		V-Set Domain Containing T Cell Activation Inhibitor 1 (VTCN1), PD-L1, CTLA4	Endometrial	Retrospective observational with bioinformatics analysis	“High methylation age deceleration linked to VTCN1-driven immunoexclusion.”	[92]
↑Methylation	PARVG, SYNE4, CDO1	↓T cells CD8, ↓Eosinophils and ↓Neutrophils, ↑M2 macrophages and ↑M0 macrophages ↓CTLA4, ↓PD1, ↓PD-L1, and ↓PD-L2	UCEC	Retrospective observational cohort study with bioinformatic	“Hypermethylation increases immunosuppression, higher TMB.”	[93]
↑Methylation	Capping actin protein, gelsolin-like (CAPG)	↓IL8, ↓TNF, ↓TLR4 ↑Plasma cells, ↑CD8 T cells, ↑follicular helper T cells, ↑M0 macrophages, ↑M1 macrophages, and ↑macrophages ↓Naive B cells, ↓resting CD4 memory T cells, ↓eosinophils, ↓resting mast cells, and ↓resting NK cells	UCEC Ishikawa	Retrospective observational cohort study with bioinformatics In vitro	“CAPG methylation impacts immune response and ferroptosis.”	[94]
↑Methylation	MutL protein homolog 1 (MLH1)	↑PD-1/PD-L1	Endometrial	Retrospective observational cohort	“MLH1 methylation enhances PD-1/PD-L1 in immune cells.”	[95]
↑Methylation ↓Methylation	↓MSX1, IL18BP ↑SLC9A1, MARVELD1, PI3, MFAP4	↑IL-18, ↓T-cell activity ↓MHC-I, ↓PD-L1, ↓LAG3	Ovarian OVCAR3	Retrospective observational cohort study with bioinformatics In vitro	“Hypomethylation promotes tumor growth, hypermethylation impairs immunity.”	[96]
↑Methylation	↑ZC3H13, YTHDC1 (no effect), and ↑METTL14	↑PD-L1, ↑CD4 + T cells, ↓macrophages, NK cells	Endometrial Ishikawa, HEC-1A	In vitro	“METTL14/ZC3H13 loss drives immune evasion via m6A reduction.”	[97]
**Acetylation**						
↑Acetylation		↑PD-L1 ↓B cells, ↓CD4 + and CD8 + T cells, and ↓cancer-associated fibroblasts (CAF)	CESC CC7T/VGH, CaSki	Retrospective observational cohort study with bioinformatics In vitro	“ACSS2 upregulation promotes immunosuppressive TIME.”	[86]
↑Acetylation	GALNT6A, DNAJA1, PRDX5, SERPINB9, FOXO1, ACSM3	M2/Tregs PD-1/PD-L1, CD8 +/M1	Ovarian	Retrospective observational cohort study with bioinformatic	“C1 pattern predicts better ICB response, C3 linked to proliferation.”	[83]
↑Acetylation	FOXP1	↑PD‐L1, CTLA4, CXCL9, ARG1 ↑Treg infiltration, ↑CD4 + T, ↓M1 infiltration	Cervical SiHa and CaSki	In vitro and in vivo	“NAT10/ac4C enhances immunosuppression via glycolytic reprogramming.”	[98]
**m6A and m1A modification**						
m6A	m6A regulators	METTL3/m6A/MDSCs, M1 to M2 macrophage polarization, IL-1β, CCL2 and CXCL2	Ovarian ID8	In vitro and in vivo	“METTL3 knockout shifts macrophages to M2, promoting tumor growth.”	[99]
m6A	m6A regulators	T-cell immunoreceptor with Ig and ITIM domains (TIGIT), CD8^+^ T cells, CD4^+^ T cells, NK cells, and Macrophage M1	UCEC	In vitro and in vivo	“TIGIT strongly tied to immune function, impacts infiltration.”	[100]
↓m6a	m6A regulators	↑HLA, ↑PD-1, ↑CTLA-4 ↑CD4 +/CD8 + T cells and dendritic cells	Ovarian	Retrospective observational with bioinformatics analysis	“Low m6A enhances immune activation, better immunotherapy response.”	[101]
↓m6a	m6A regulator: RBM15	↓NK cell, ↑PD-L1	UCEC	Retrospective observational with bioinformatics analysis	“RBM15-mediated m6A inhibits NK cell activation, promotes PD-L1.”	[102]
m1A	m6A regulators	CTLA4, PD-1	Ovarian	Retrospective observational with bioinformatics	“High m1A score predicts better anti-PD-1/CTLA-4 response.”	[103]
↑m6A		↓CTLA-4, ↓PD-1, and ↓PD-L1	CESC	Retrospective observational with bioinformatics	“Low m6A score linked to higher TMB, better immunotherapy response.”	[104]

### Acetylation

Within mammalian cells, DNA is tightly packaged into chromatin, wrapped around histone proteins that undergo various posttranslational modifications. Among these, acetylation of lysine residues on histones H3 and H4 is a key regulator. This process, driven by histone acetyltransferases (HATs), adds acetyl groups, loosening chromatin structure and enhancing gene accessibility at promoter or enhancer regions, thereby promoting gene expression. Conversely, HDACs remove these acetyl groups, condensing chromatin and silencing gene activity [76]. This dynamic interplay between HATs and HDACs shapes the epigenetic landscape, influencing immune-related gene expression and the tumor microenvironment in gynecological cancers. In gynecological cancers, including cervical and ovarian carcinomas, acetylation orchestrates epigenetic and metabolic pathways that shape tumor progression and immune responses [77, 78]. By modulating the TIME and immune checkpoint pathways, acetylation significantly impacts immunotherapy efficacy**,** particularly through regulating PD-L1 expression, immune cell infiltration, and tumor cell survival, positioning it as a promising target for therapeutic innovation [79].

In cervical cancer, acetylation drives epigenetic mechanisms that influence immunotherapy outcomes. The nicotinamide adenine dinucleotide (NAD +)-dependent deacetylase Sirtuin 1 (SIRT1), within the NAMPT/SIRT1 metabolic axis, deacetylates H3K27, modulating PD-L1 nuclear localization and expression. This axis contributes to resistance against immune checkpoint inhibitors, underscoring acetylation’s pivotal role in immunotherapy strategies [80]. Additionally, long non-coding RNA (lncRNA)-encoded peptides, such as TUBORF, are acetylated by enzymes like ESCO1, promoting malignant proliferation by suppressing ferroptosis, a programmed cell death pathway. Human papillomavirus (HPV) oncoproteins E6 and E7 amplify this process by enhancing H3K27 acetylation through CBP/p300, upregulating lncTUBA3FP and ESCO1 expression. This cascade not only drives tumorigenesis but also fosters immune evasion by stabilizing oncogenic peptides, linking acetylation to immunotherapy resistance and tumor aggressiveness [81, 82]. 

In epithelial ovarian carcinoma (EOC), acetylation patterns profoundly influence immune cell dynamics and therapeutic responses [9]. Gene expression studies categorize acetylation-related genes into three patterns (C1, C2, C3), each with distinct implications for TIME. The C1 pattern, enriched in immune-related pathways, associate with enhanced responsiveness to PD-1 blockade, highlighting acetylation’s potential as a predictive biomarker for immunotherapy patient stratification**.** In contrast, the C3 pattern is associated with tumor proliferation pathways, such as Hedgehog signaling, which is linked to poorer prognosis and increased malignancy. Acetylation also regulates immunosuppressive cells, including tumor-associated macrophages (TAMs) and regulatory T cells (Tregs). For instance, inhibitors like berberine (BBR) reduce p65 acetylation, suppressing inflammatory responses in TAMs, while NAC1 destabilizes FoxP3 acetylation in Tregs, promoting tumorigenesis. Low-risk EOC groups, characterized by high T cell and M1 macrophage infiltration, benefit from acetylation-driven immune activation, enhancing immunotherapy outcomes and connecting epigenetic regulation to immune surveillance. Acetylation also influences chemotherapy and immunotherapy resistance, offering insights into combination therapies. In EOC, acetylation patterns predict sensitivity to chemotherapeutic agents like cisplatin and paclitaxel, with distinct IC50 values across C1, C2, and C3 clusters. Genes such as FOXO1, a risk factor, and GALNT6 contribute to chemoresistance by modulating immune cell dynamics, while protective factors like ACSM3 inhibit tumor proliferation. In cervical cancer, targeting acetylation-related pathways, such as the NAMPT/SIRT1 axis or ESCO1, enhances paclitaxel sensitivity by increasing ferroptosis, suggesting synergy with immunotherapy. Acetylation’s critical influence on immunotherapy is evident in its ability to stratify patients for immune checkpoint blockade therapies, with C1 patterns and high tumor mutational burden (TMB) indicating improved responses. This predictive capacity guides precision medicine, enabling tailored treatments for gynecological cancer patients [83–85]. Metabolic reprogramming, a hallmark of gynecological cancers, is intricately linked to acetylation, further shaping TIME. The Warburg effect, marked by elevated glycolysis, supports rapid tumor growth, with acetylation modulating key metabolic enzymes like ACSS2. In cervical squamous cell carcinoma (CESC), ACSS2 upregulation fosters an immunosuppressive TIME by enhancing cancer-associated fibroblast (CAF) exhaustion and Treg activity, promoting tumor invasion and metastasis. This metabolic-epigenetic interplay emphasizes acetylation’s role in immunotherapy efficacy**,** as it regulates immune checkpoint receptor expression and immune cell functionality [86]. In ovarian cancer, acetylation of p53 by P300 enhances its stability and transcriptional activity, inducing cellular senescence and inhibiting tumor growth, as observed with CDK4/6 inhibitors like Palbociclib. This mechanism not only curbs tumor progression but also amplifies immune-modulatory pathways, reinforcing acetylation’s multifaceted role in cancer control [87]. 

Acetylation emerges as a cornerstone of epigenetic regulation in gynecological cancers, intricately Linking chromatin remodeling, metabolic reprogramming, And immune modulation. As indicated in Table 1, its ability to shape TIME, regulate immune checkpoint pathways, an influence therapeutic responses underscores its potential as both a biomarker and therapeutic target. By driving immunotherapy efficacy, acetylation offers a pathway to overcome resistance and enhance patient outcomes, particularly through personalized strategies informed by acetylation patterns. Future research should focus on elucidating the molecular mechanisms underlying acetylation’s interactions with TMB and TIME, validating these findings in larger clinical cohorts, and developing targeted inhibitors, such as those for SIRT1 or ESCO1, to complement ICB therapies. As precision oncology advances, harnessing acetylation’s regulatory power promises to transform the therapeutic landscape for cervical and ovarian cancers, paving the way for more effective and individualized treatments.

### Histone methylation

Histone methylation serves as a critical epigenetic mechanism, distinct from histone acetylation, that intricately regulates gene expression through the addition or removal of methyl groups on specific histone residues. This process varies in complexity depending on the targeted amino acid and the degree of methylation [105]. For example, trimethylation of lysine 4 on histone 3 (H3K4me3) is typically linked to active promoters or enhancers, promoting an open euchromatin structure that facilitates gene transcription. Conversely, monomethylation of H3K4 (H3K4me1) marks poised enhancers, while H3K27me3 fosters a condensed heterochromatin state, silencing gene expression [106]. These modifications are tightly controlled by histone methyltransferases (HMTs), which add methyl groups, and histone demethylases (HDMTs), which remove them. Over 60 unique HMTs and HDMTs have been identified, underscoring the diversity and precision of this regulatory system in shaping the TIME and influencing immunotherapy outcomes in gynecological cancers. Dysregulated histone methylation, particularly at specific lysine residues like H3K4, H3K9, and H3K27, disrupts the expression of genes critical for immune recognition and response, creating an immunosuppressive TIME that hinders effective immunotherapy [107, 108]. Understanding these mechanisms offers promising avenues for targeted therapies to enhance antitumor immunity in gynecological malignancies.

In cervical cancer, histone methylation significantly shapes the TIME and immunotherapy potential through mutations in epigenetic regulators like MLL2, a histone methyltransferase that methylates H3K4. Studies have shown that MLL2 mutations are associated with poor survival, likely due to aberrant H3K4 methylation, which alters the epigenetic landscape and promotes cancer progression. These mutations disrupt normal histone modification patterns, potentially suppressing genes involved in immune activation. Additionally, mutations in other epigenetic regulators, such as EP300, CREBBP, and SETD2, often occur near phosphorylation sites, further dysregulating histone methylation and chromatin condensation. This dysregulation impairs antigen presentation and immune cell recruitment, creating an immunosuppressive TIME. Notably, the immunogenicity of mutations in cervical cancer, driven by altered histone methylation, generates neo-epitopes that can be recognized by T cells, suggesting that targeting these epigenetic changes could enhance immunotherapy efficacy. For instance, Chromatin Immunoprecipitation followed by sequencing (ChIP-seq) for H3K4me3 could identify specific methylation patterns to guide immunotherapeutic strategies [109]. Moreover, m5C RNA methylation, regulated by NSUN2 and YBX1, complements histone methylation by stabilizing oncogenic mRNAs, further suppressing immune responses. The negative correlation between m5C scores and PD-L1 expression, alongside a positive association with tumor mutation burden (TMB), underscores the potential of combining epigenetic therapies with immune checkpoint inhibitors to improve immunotherapy responses in cervical cancer [110].

In ovarian cancer, histone methylation regulates the TIME and immunotherapy efficacy through the actions of methyltransferases like SETDB1 and demethylases like KDM5A [111, 112]. SETDB1, a H3K9 methyltransferase, is overexpressed in ovarian cancer and promotes tumor progression by silencing tumor suppressor genes and inhibiting antitumor immune responses. By interacting with the SF3B4 promoter, SETDB1 enhances oncogenic gene expression, while its amplification reduces immune cell infiltration and PD-L1 expression, fostering an immunosuppressive TIME [111]. Similarly, KDM5A, a H3K4 demethylase, impairs CD8 + T-cell infiltration by silencing genes involved in antigen processing and presentation, such as HLA-A and HLA-B. Inhibiting KDM5A restores these genes’ expression, enhancing CD8 + T-cell-mediated antitumor immunity, as demonstrated in syngeneic mouse models. This highlights the therapeutic potential of targeting histone methylation to boost immunotherapy in ovarian cancer [112]. Furthermore, SETDB1’s role in repressing the cGAS-STING pathway and CD8 + T-cell infiltration suggests that its downregulation could enhance radiotherapy and immunotherapy outcomes [111]. The interplay between histone methylation and immune evasion underscores the need for integrated approaches combining epigenetic inhibitors with immunotherapies to overcome resistance and improve survival in ovarian cancer patients.

The interconnected roles of histone methylation in cervical and ovarian cancers highlight its broader significance in gynecological malignancies. In both cancers, dysregulated histone methylation contributes to immune evasion by altering the TIME**,** suppressing antigen presentation, and reducing immune cell infiltration. However, the reversibility of epigenetic modifications makes histone methylation a promising target for immunotherapy**.** For cervical cancer, therapies targeting MLL2 or m5C regulators like NSUN2 could restore immune recognition, while in ovarian cancer, inhibiting SETDB1 or KDM5A could enhance T-cell responses. The shared reliance on histone methylation to shape the TIME suggests that combination therapies, integrating epigenetic modulators with immune checkpoint inhibitors, could synergistically improve clinical outcomes. Future research should focus on large-scale genomic analyses to map histone methylation patterns across clinical stages and explore interactions with other epigenetic modifications, such as DNA and RNA methylation, to develop personalized immunotherapeutic strategies for gynecological cancers.

### Histone lactylation

Histone lactylation, a novel epigenetic modification involving the attachment of lactate to lysine residues on histones, has emerged as a critical regulator of gene expression in cancer, particularly in the TIME [113]. This process, driven by lactate produced through enhanced glycolysis in tumor cells, modulates immune responses and influences the efficacy of immunotherapies. Histone lactylation plays a pivotal role in immunotherapy by epigenetically regulating the expression of immune checkpoint molecules, such as PD-L1, thereby affecting T-cell activation and tumor immune evasion [114, 115]. In gynecological cancers, such as ovarian and cervical cancer, lactate-driven histone lactylation shapes the TIME by promoting immunosuppressive mechanisms, enhancing tumor aggressiveness, and altering immune cell infiltration [116, 117]. Understanding these mechanisms offers promising avenues for developing targeted immunotherapeutic strategies that disrupt lactate-mediated epigenetic regulation.

In ovarian cancer, histone lactylation significantly influences immunotherapy outcomes by regulating PD-L1 expression through metabolic and epigenetic pathways. Lactate dehydrogenase B (LDHB) enhances glycolysis and lactate production, leading to increased histone lactylation at the PD-L1 promoter, which upregulates PD-L1 expression. This upregulation suppresses T-cell activation, facilitating immune evasion by ovarian cancer cells [116]. Studies have shown that knocking down LDHB reduces glucose metabolism and lactate accumulation, subsequently decreasing histone lactylation and PD-L1 expression [118]. Conversely, PD-L1 overexpression can counteract LDHB inhibition, underscoring the critical role of this pathway. The interplay between LDHB, lactate, and histone lactylation highlights a metabolic-epigenetic axis that could be targeted to enhance the efficacy of ICIs. For instance, combining ICIs with therapies that inhibit LDHB or lactate production may restore T-cell cytotoxicity, offering a novel strategy to overcome immune suppression in ovarian cancer [116]. In cervical cancer, histone lactylation drives immunosuppression and tumor progression within the TIME by modulating immune cell function and gene expression. Tumor-secreted lactate, a byproduct of aerobic glycolysis, accumulates in the TIME, promoting histone lactylation and altering the expression of genes involved in immune regulation [117]. Notably, the downregulation of EP300, a histone acetyltransferase, in cervical cancer reduces histone lactylation, stabilizing cancer cell proliferation and inhibiting anti-tumor immune responses [119]. Elevated lactate levels, driven by enzymes like LDHA and monocarboxylate transporters (MCTs), correlate with increased tumor aggressiveness, angiogenesis, and poor survival. Genes such as LDHA, ACACA, and SLC16A3 are upregulated in cervical cancer, contributing to lactate-mediated epigenetic changes that suppress DC and T-cell activity. For example, high lactic acid levels hinder plasmacytoid DC and central memory T-cell accumulation, fostering an immunosuppressive TIME. Targeting histone lactylation through inhibitors of LDHA or MCTs may reduce immunosuppression and enhance the effectiveness of ICIs in cervical cancer [117].

The broader implications of histone lactylation extend to the metabolic adaptability of gynecological tumors. Tumor cells exploit anaerobic glycolysis and lactate fermentation, catalyzed by LDHA and LDHB, to thrive in hypoxic conditions. This metabolic flexibility not only supports energy production but also drives epigenetic changes that enhance immune evasion. In cervical cancer, the lactate shuttle enables aerobic cancer cells to utilize lactate from hypoxic cells, further amplifying histone lactylation and gene expression changes. Similarly, in ovarian cancer, lactate-induced STING-dependent innate immune responses and PD-L1 upregulation create a feedback loop that sustains immunosuppression. By targeting histone lactylation**,** therapies could disrupt this feedback loop, restoring immune surveillance and enhancing the efficacy of immunotherapies. Future research should explore additional regulatory mechanisms, such as the role of STAT5 in promoting glycolysis and lactylation, to fully elucidate the therapeutic potential of targeting lactate metabolism in gynecological cancers.

### N6-methyladenosine and N1-methyladenosine

RNA modifications, notably N6-methyladenosine (m6A) and N1-methyladenosine (m1A), are key epigenetic regulators of gene expression and cellular processes in gynecological cancers. These modifications, part of the epitranscriptome, involve the addition of chemical groups to RNA nucleotides, modulating post-transcriptional regulation [120, 121]. Over 170 RNA modifications have been documented, with m6A and m1A being particularly influential due to their effects on RNA stability, translation, and interactions with cellular machinery. This process is governed by three protein classes: writers, which add modifications; erasers, which remove them; and readers, which bind to modified RNAs [122]. For m6A, writers like METTL3, METTL14, and WTAP drive methylation, erasers such as FTO and ALKBH5 reverse it, and readers including YTHDF1 and IGF2BP1 mediate downstream effects. For m1A, writers such as TRMT6 and TRMT61A, erasers like ALKBH1 and ALKBH3, and readers like YTHDF2 regulate the modification. In ovarian cancer, RNA-modification regulatory genes (RRGs) are critical drivers of tumorigenesis and drug resistance, with ALKBH5 promoting cancer progression through the NF-κB pathway and IGF2BP3 contributing to platinum resistance, highlighting their role in shaping cancer biology and therapeutic responses [123].

The tumor TIME in gynecological cancers is profoundly influenced by m6A and m1A modifications, which regulate immune cell infiltration and activation. In ovarian cancer, m6A regulators such as METTL3 and ALKBH5 modulate immune checkpoint expression, including PD-L1, impacting T-cell function and infiltration. ALKBH5, for instance, upregulates PD-L1 in macrophages, suppressing immune effector cells within the TIME [124]. Similarly, m1A modifications in ovarian cancer define distinct immune profiles, ranging from immune-inflamed to immune-desert phenotypes. The m1A cluster-B, characterized by elevated CD4 + T-cell and myeloid cell infiltration, supports adaptive immunity, whereas cluster-C reflects immune tolerance. These patterns directly affect immunotherapy outcomes, particularly with ICIs. The interplay between RNA modifications and TIME underscores their potential as therapeutic targets, offering insights into optimizing immunotherapy strategies across gynecological cancers [103]. In cervical cancer, m6A modifications further illuminate their impact on TIME and immunotherapy potential [125]. Research indicates that m6A regulators like METTL3, METTL14, and YTHDF1 are overexpressed in cervical tumors compared to normal tissues, with elevated METTL3 and YTHDF1 levels linked to poorer prognosis [126]. These regulators drive tumor progression and influence immune infiltration, with YTHDF1 promoting Treg infiltration, potentially suppressing Anti-tumor immunity. Molecular subtyping reveals two clusters, with cluster 2 showing increased plasma cell and Treg presence, associated with lower tumor stage but no significant survival difference. The association of m6A regulators with PD-L1 expression suggests their potential as predictors of immunotherapy response, calling for further research to refine treatment approaches [125]. In endometrial cancer, the second most common gynecological malignancy after cervical cancer, m6A modifications play a significant role in tumor progression and immune regulation [127]. With rising global incidence, endometrial cancer’s immune landscape is shaped by m6A patterns, as demonstrated by studies involving 1,301 tumor samples. Analysis of 21 m6A regulators revealed four distinct modification classes, each with unique immune infiltration profiles. Class 2 And class 3, characterized by adaptive immune cell infiltration And immune activation, attribute with better survival, while class 1 And class 4, linked to stromal activation and immunosuppression, indicate poorer prognosis. A scoring system, termed m6Ascore, quantifies these patterns, with lower scores associated with immune-inflamed phenotypes and improved outcomes, and higher scores tied to immune-desert phenotypes. Genetic analyses show frequent m6A regulator mutations (33.21% of samples) and copy number variations, particularly amplifications, with YTHDF1 and YTHDF2 showing deletions. Notably, m6Ascore predicts immunotherapy response, with low scores linked to enhanced anti-PD-1/PD-L1 efficacy, suggesting m6A modifications as valuable biomarkers for tailoring endometrial cancer treatments [102, 128].

The therapeutic potential of targeting m6A and m1A modifications extends to improving drug sensitivity and immunotherapy outcomes in gynecological cancers. In ovarian cancer, RRGs like DNMT1 and METTL3 are implicated in resistance to cisplatin and paclitaxel, while m1A regulators such as TRMT10C influence tumor proliferation. Small-molecule drugs, including resveratrol and amodiaquine, show preclinical promise by targeting these modifications to inhibit tumor growth and enhance immune responses. In cervical cancer, a prognostic signature comprising METTL16, YTHDF1, and ZC3H13 predicts survival and immunotherapy response, with high-risk scores indicating worse outcomes. Similarly, in endometrial cancer, m6Ascore serves as an independent prognostic indicator, aiding patient stratification for immunotherapy. By integrating molecular signatures with clinical data, m6A and m1A modifications provide a robust framework for precision oncology, enabling personalized therapies that harness epigenetic regulation to optimize immunotherapy efficacy across gynecological cancers.

## Epigenetic regulators in shaping the tumor immune microenvironment and immunotherapy response in gynecological cancers

### DNMT and DNMT inhibitors

DNMTs are pivotal epigenetic regulators that orchestrate gene expression by catalyzing the methylation of cytosine residues in CpG dinucleotides, a process integral to cellular differentiation And development. Since their discovery in the 1970s, three primary DNMT isoforms—DNMT1, DNMT3A, and DNMT3B—have been extensively studied [18]. DNMT1 ensures the maintenance of methylation patterns during DNA replication, preserving epigenetic memory, while DNMT3A and DNMT3B establish de novo methylation, shaping gene expression profiles during embryogenesis and disease states [129]. In gynecological cancers, including ovarian and endometrial carcinomas, dysregulated DNMT activity drives oncogenesis by hypermethylating tumor suppressor genes, such as BRCA1, and hypomethylating oncogenes, promoting uncontrolled proliferation. Within the TIME, DNMTs suppress immune surveillance by silencing genes encoding MHC class I molecules, chemokines, and cancer-testis antigens (CTAs), thereby enabling immune evasion [129]. DNMTis, including azacitidine, decitabine, and guadecitabine (SGI-110), have revolutionized immunotherapy approaches by reversing these epigenetic alterations, enhancing antigen presentation, and invigorating immune cell recruitment to combat gynecological malignancies effectively (Table 2).

**Table 2:** Effects of HDAC and DNMT inhibitors on immune modulation of gynecological cancers

Epigenetic regulator	Immune component targets	Cancer types and cell lines	Highlights	Ref
HDAC1i + DNMT1i	↑4-1BB ligand (4-1BBL/CD157) and OX-40 ligand (OX-40L/CD252) ↓PD-L1/CD274	Ovarian A2780	“Knockdown of HDAC1 or DNMT1 expression, and pharmacological inhibition of DNMT or HDAC enzymatic activity, significantly increase OX-40L And 4-1BBL expression in chemoresistant cells. This study suggests that loss of histone acetylation and accumulation of DNA methylation correlates with suppressed expression of OX-40L And 4-1BBL in chemoresistant ovarian cancer cells, assosiat with promoted cytotoxic T-cells activity.”	[15]
HDACi + DNMTi	↑CIITA, CD74, and MHC II	Ovarian A2780.IP2 and A2780.CP20	“Combination treatment showed higher MHC II protein expression than with single agent treatment. In patient-derived xenografts, CIITA, CD74, and MHC II mRNA transcripts were significantly increased after combination treatment.”	[151]
HDACi + DNMTi	↑CCL5, TNF-α and IFN-γ ↓IL-10	Ovarian OVCAR-3, SKOV-3, A2780	“The treated bulk T cells, CD8 + T cells, and CD4 + T cells showed anti-tumor cytokines, including CCL5, TNF-α and IFN-γ, were upregulated while the pro-tumor cytokine IL-10 was downregulated. These changes were evident at the transcript and secreted cytokine level, pointing to transcriptional upregulation of the target genes. This study demonstrates direct activation of T cells by DNMTis and HDACis and provides mechanistic insight into the role of epigenetic therapies in the murine model.”	[152]
HDACi	↓IKK/↑IL-8/CXCL8	Ovarian SKOV3, CAOV3, and OVCAR3	“HDAC inhibition specifically induces IL-8/CXCL8 expression in EOC cells and that the mechanism involves IKK, suggesting that using IKK inhibitors may increase the effectiveness of HDAC inhibitors when treating ovarian cancer and other solid tumors characterized by increased IL-8/CXCL8 expression.”	[153]
HDACi	↓IL-6, IL-8, IL-1B and CXCL1	Ovarian In vivo	“ARID1A recruits the Sin3A-HDAC complex to suppress cytokine gene expression. HDAC inhibitors would not be effective for OCCC, and could even cause unfavorable outcomes.”	[154]
HDACi	↑NKT, and CD8^+^ T cells ↑PSMB10, NKG7, ↑CCL5, ↑CD27, ↑HLA-DQA1, and HLA-DQB1	Ovarian In vivo	“Increased HDAC expression was linked to reduced infiltration of natural killer (NK), NKT, and CD8^+^ T cells, along with negative associations with the expression of PSMB10, NKG7, CCL5, CD27, HLA-DQA1, and HLA-DQB1. In a murine 4T1 model, treatment with suberoylanilide hydroxamic acid (SAHA; HDAC inhibitor) and PD-1 antibody significantly inhibited tumor growth and infiltration of CD3^+^ and CD8^+^ T cells.”	[16]
HDACi	KANSL1	Ovarian	“Histone acetylase and methylation complex gene *KANSL1* May lead to hypermethylation of 6p21 resulting in an inverse correlation of mRNA expression of the prognostic immune genes mapping to this region that may control T-cell infiltration; thus Targeting KANSL1 may alter immune profile of ovarian cancer, improve survival, HDAC inhibition, andimmunotherapy response.”	[155]
DNMTi	↑Cytotoxic CD8 + T, ↑type I IFN	Ovarian A2780, SKOV3, Kuramochi	“Combining Adar1 loss and DNMTi elicited the most robust antitumor response and transformed the immune microenvironment with increased recruitment and activation of tumor-specific, cytotoxic CD8 + T cells.”	[156]

In ovarian cancer, DNMTis profoundly reshape the TIME, fostering an immunogenic landscape conducive to anti-tumor responses. Research demonstrates that SGI-110 induces hypomethylation of CTA promoters, such as NY-ESO-1 and MAGE-A, in EOC cell lines and tumor-bearing murine models, resulting in robust mRNA and protein expression. This upregulation enhances tumor cell recognition by antigen-specific CD8 + T cells, particularly when paired with NY-ESO-1-directed vaccines, leading to delayed tumor growth compared to standalone treatments [19]. Moreover, DNMTis like azacitidine amplify interferon-gamma (IFNγ) signaling, promoting M1 macrophage polarization and increasing tumoricidal activity. A notable study combining azacitidine with difluoromethylornithine in immunocompetent ovarian cancer mouse models revealed significant reductions in tumor burden and extended survival. This regimen boosted IFNγ + immune cell populations, including natural killer (NK) cells, CD4 + T cells, and CD8 + T cells, while enhancing M1 macrophage prevalence through IFNγ-driven polarization and arginine metabolism modulation. Depletion of macrophages using CSF1R antibodies diminished these therapeutic benefits, underscoring their tumoricidal role. These findings highlight DNMTis’ capacity to convert immunosuppressive microenvironments into immunologically active ones, paving the way for synergistic immunotherapies in ovarian cancer [130].

The immunomodulatory potential of DNMTs extends to their interplay with RNA editing enzymes, notably adenosine deaminase acting on RNA 1 (ADAR1), offering novel therapeutic avenues [131, 132]. DNMTi treatment, when combined with ADAR1 inhibition, activates type I IFN signaling by increasing immunogenic double-stranded RNA (dsRNA), which triggers the production of chemokines like CCL5 and CXCL10. This cascade transforms the TIME, markedly enhancing CD8 + T cell infiltration and NK cell cytotoxicity, resulting in significant tumor burden reduction and prolonged survival in aggressive, clinically relevant murine models [133]. Importantly, DNMTis sensitize TP53-mutant ovarian cancer cells—present in over 90% of high-grade serous ovarian carcinomas to IFN responses, amplifying anti-tumor immunity across diverse TP53 mutational statuses. The mechanistic nuances between azacitidine, which integrates into both DNA and RNA, and DAC, which targets only DNA, suggest differential impacts on RNA editing, splicing, and transcriptional regulation. For instance, azacitidine’s RNA incorporation may disrupt ADAR1-mediated editing, further amplifying dsRNA immunogenicity. These insights emphasize DNMTis’ multifaceted roles in immunotherapy, not only as epigenetic modulators but also as enhancers of innate immune sensing, broadening their applicability in ovarian cancer treatment [134].

In endometrial cancer, particularly MMRd subtypes, DNMTis address TIME heterogeneity to optimize ICI efficacy [135]. MMRd endometrial cancers harbor high neoantigen loads, predisposing them to T cell recruitment; however, variable lymphocyte infiltration often limits ICI success [136]. Spatial transcriptomic analyses reveal that elevated DNMT3A expression correlates with reduced CD8 + T cell infiltration and diminished HLA class I expression, fostering immune evasion. DNMTis counteract this by upregulating HLA class I and chemokines like CCL5, promoting a “hot” TIME characterized by robust CD8 + T cell presence. A 14-gene signature, encompassing HLA class I genes, stratifies MMRd endometrial cancers into hot, intermediate, and cold immune subtypes, with DNMTi-induced HLA class I upregulation emerging as a promising biomarker for ICI candidate selection. However, the presence of M2-like macrophages and Tregs in hot tumors can dampen anti-tumor effects, highlighting the need for combination therapies to neutralize immunosuppressive elements. For instance, integrating DNMTis with therapies targeting Treg recruitment could enhance ICI outcomes. Additionally, DNMT3A’s negative regulation of CCL5 and its receptors, CCR1 and CCR5, suggests a broader epigenetic control over T cell trafficking, further underscoring DNMTis’ potential to fine-tune TIME dynamics in endometrial cancer [135].

The cohesive impact of DNMTis across gynecological cancers lies in their ability to reprogram the TIME through interconnected mechanisms: enhancing CTA and HLA class I expression, amplifying IFN signaling, polarizing macrophages toward tumoricidal phenotypes, and boosting chemokine-driven immune cell infiltration. By linking innate and adaptive immunity, DNMTis synergize with vaccines, ICIs, and emerging targets like ADAR1 inhibitors, offering a versatile platform for combination therapies. Their efficacy in overcoming immune evasion, particularly in TP53-mutant ovarian cancers and MMRd endometrial cancers, positions DNMTis as cornerstone agents in personalized immunotherapy. Future research should explore optimal DNMTi combinations with other epigenetic modifiers, such as histone deacetylase inhibitors, to further potentiate immunogenicity and address TIME immunosuppression comprehensively. Ultimately, DNMTis represent a transformative force in gynecological cancer immunotherapy, bridging epigenetic regulation with immune activation to improve clinical outcomes.

### HDAC and HDAC inhibitors

HDACs are a family of enzymes critical to epigenetic regulation, governing gene expression by removing acetyl groups from histone proteins, thereby condensing chromatin and typically suppressing transcription [137]. In the context of gynecological cancers, such as cervical and ovarian cancers, HDACs play a pivotal role in promoting tumorigenesis by silencing tumor suppressor genes, enhancing cell proliferation, migration, and angiogenesis. Their influence extends beyond epigenetics into the TIME, where they modulate immune responses by regulating the expression of MHC molecules, antigen presentation, and immune cell activity [10]. HDACi have emerged as a transformative therapeutic strategy in immunotherapy, counteracting these effects by promoting histone acetylation, which reactivates suppressed genes, enhances tumor antigen presentation, and bolsters immune recognition, offering a promising avenue to improve antitumor immunity in gynecological malignancies [138].

In cervical cancer, studies utilizing the TC-1 tumor model demonstrate that combining HDACi, such as suberoylanilide hydroxamic acid (SAHA, or Vorinostat), with bortezomib, a proteasome inhibitor, yields synergistic antitumor effects. This combination significantly increases E7-specific CD8 + T-cell populations in the spleen, circulation, and tumor, enhancing immune activation and rendering tumor cells more susceptible to T-cell-mediated killing [139]. Similarly, valproic acid alone or with hydralazine boosts CTL recognition by upregulating HLA class I in CaSki and MS751 cells, exceeding single-drug effects and slightly enhancing IFN-γ’s impact, showing acetylation’s dominance over methylation. Unlike sporadic de novo HLA expression seen elsewhere, cervical lines showed basal HLA and processing components, with hydralazine/valproic acid enhancing CTL recognition of HPV E6/E7 peptides, peaking with VPA or combination, suggesting acetylation drives recognition over IFN-γ. This first study of hydralazine/valproic acid in cervical cancer shows HLA and CTL boosts, with potential to reactivate antigens like MAGE/GAGE and NKG2D ligands, linking to ovarian strategies enhancing immunotherapy via TIME modulation [140, 141]. HDACi’s role in immunotherapy is underscored by their ability to amplify tumor-specific immunity through enhanced antigen release and cross-priming by antigen-presenting cells (APCs), linking direct tumor cell targeting with robust immune activation, a synergy that holds potential for combination with therapeutic vaccines like CRT/E7 DNA [139].

Transitioning to ovarian cancer, HDACi demonstrate parallel immune-modulatory effects within the TIME (Table 2). In spontaneous (MISIIR) and aggressive (ID8) murine EOC models, class I HDAC inhibitors like entinostat upregulate tumor antigen expression and MHC class II pathways, driven by IFNγ signaling. This upregulation fosters a more inflamed microenvironment, conducive to CD8 + T-cell activation. Furthermore, entinostat treatment reduces the expression of CXCL1-5, suppressive myeloid chemoattractants that potentially limit MDSC accumulation. It also decreases Treg frequency and FoxP3 expression, thereby alleviating immunosuppression. The contribution of HDAC inhibitors to immunotherapy is evident in the increased CD8 + T-cell-to-Treg ratio and improved Tbet + CD8 + T-cell persistence. This indicates a shift toward a pro-inflammatory TIME; however, optimal efficacy may require earlier intervention or combination with other immune stimulants to counter T-cell exhaustion in established tumors. [142]. Further exploring cancers, HDACi’s regulation of chemokine CXCL8 offers a nuanced perspective [143]. While class I HDAC inhibition induces CXCL8 expression in ovarian cancer cells, potentially promoting proliferation and angiogenesis, inhibition of CBP HAT activity suppresses it, suggesting a combinatorial approach to mitigate adverse effects. This complexity ties into broader immunotherapeutic strategies, such as HLA ligandomics, where HDAC1-derived peptides identified on ovarian tumor samples stimulate multifunctional CD8 + T-cell responses in vitro, despite their absence among TILs in vivo, possibly due to immune escape or thymic tolerance. HDACi’s pivotal role in immunotherapy is highlighted by its potential to enhance peptide vaccination efficacy, particularly when paired with checkpoint inhibitors, by overcoming inadequate antigen presentation and immunosuppressive barriers within the TIME [144, 145]. Approving immune checkpoint inhibitors highlights immunotherapy’s success, yet identifying relevant targets for tumor-specific responses, like peptide vaccination, remains challenging [146].

Researchers combined HLA ligandomics with immunogenicity analysis on serous ovarian cancer (OvCa) samples and found few consistently presented proteins, primarily housekeeping genes, along with HDAC1. HDAC1 promotes tumor growth by suppressing tumor suppressor genes. Four HDAC1/2 peptides—EYSKQMQRF (HLA-A24), LPHAPGVQM (HLA-B07), RMLPHAPGV, and YTTDRVMTV (HLA-A02)—were identified, with A02 peptides present in HLA-A*02-negative patients, possibly through HLA-E presentation. HLA ligandome comparison revealed that one-third of benign ovarian sample proteins were also found in all OvCa samples, mostly housekeeping genes. HDAC1 peptides RMLPHAPGV and EYSKQMQRF appeared infrequently in benign samples but often in malignant ones, supporting the targeting of HDAC1, particularly as ovaries are resected during OvCa surgery. In vitro, LPHAPGVQM and RMLPHAPGV primed CD8 + T cells to lyse HDAC1 + OvCa cells, with RMLPHAPGV exhibiting multifunctionality, although it was absent in TILs from three OvCa patients, possibly due to immune escape or low affinity, indicating a need for peptide vaccination. Prior peptide vaccines (e.g., NY-ESO-1, p53) elicited immune responses but lacked clinical benefit, likely due to inadequate antigen selection based on gene expression rather than HLA presentation. NY-ESO-1, expressed in 43% of OvCa, has no detected HLA ligands. In refining this, researchers analyzed the OvCa HLA ligandome for universal peptides. HDAC1 overexpression was linked to worse survival, offset by high CD3 + TILs, where HDAC1 was rare, supporting its vaccine potential [147]. Finally, integrating HDACi with CAR T-cell therapy amplifies their immunotherapeutic promise in ovarian cancer. VPA upregulates NKG2D ligands on ovarian cancer cells, enhancing their recognition by NKG2D CAR T-cells, which incorporate CD137 (4-1BB) costimulation for improved persistence and cytotoxicity. Despite challenges like transient fratricide among CAR T-cells due to NKG2DL expression on activated T-cells, prolonged culture enriches functional CAR T-cells capable of robust tumor killing. HDACi enhances immunotherapy critically here, as it selectively boosts NKG2DL expression on malignant cells without affecting normal ovarian epithelium, offering a targeted strategy to overcome low antigen expression and improve CAR T-cell efficacy, potentially via regional delivery to minimize off-target effects. [148].

**Fig. 1:**
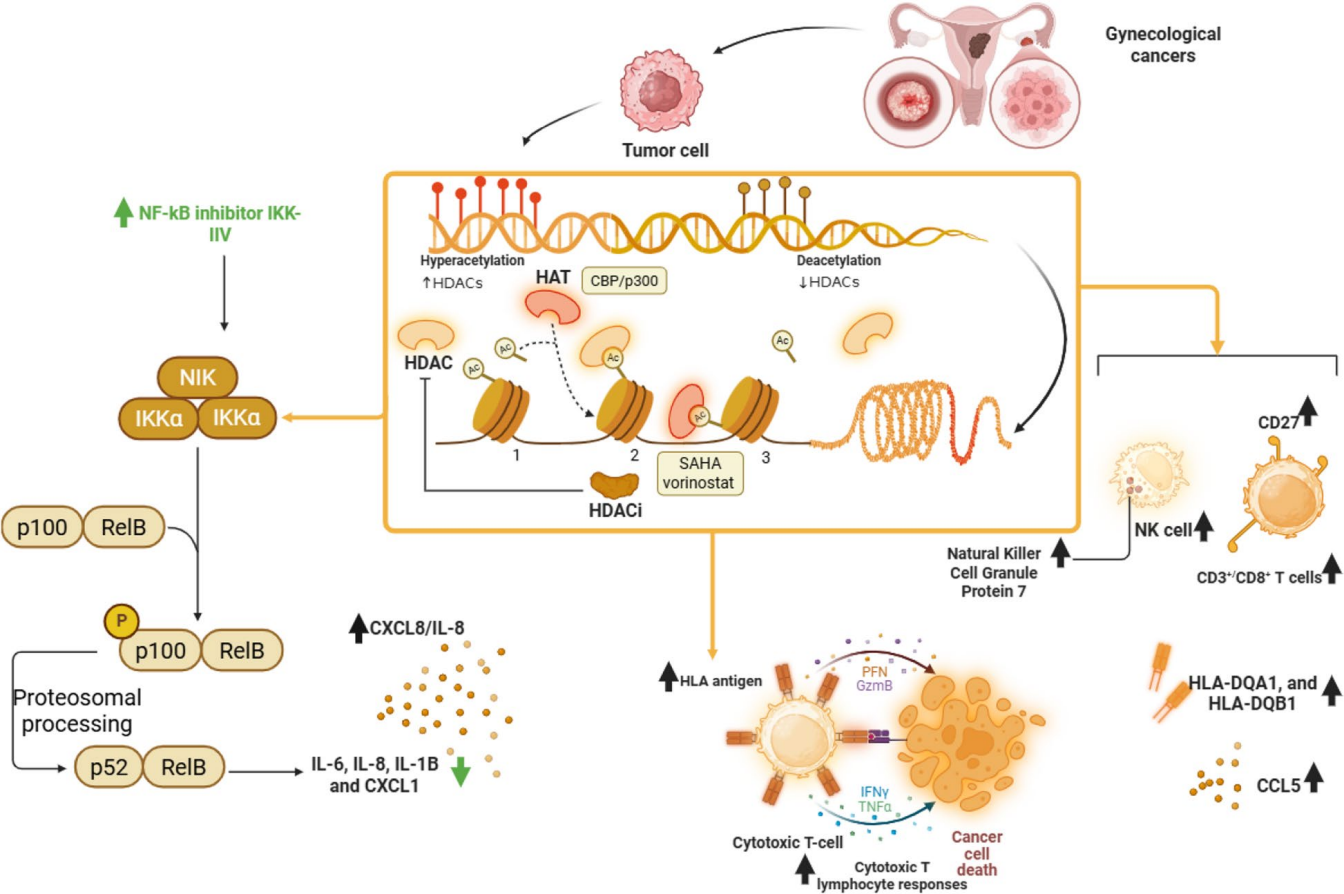
HDAC-mediated epigenetic regulation and its immunomodulatory effects in gynecological cancers. HDACs suppress gene expression by removing acetyl groups from histones, contributing to immune evasion and tumor progression. HDACi, such as SAHA/vorinostat, restore acetylation, enhancing tumor antigen presentation and immune recognition. This leads to upregulation of HLA molecules, stimulation of CTL responses, and activation of NK and CD8⁺ T cells. HDACi also alter chemokine profiles, suppress pro-tumor cytokines, IL-6, IL-8, and promote antitumor immunity, thereby reshaping the tumor immune components and potentiating the effects of immunotherapies in cervical and ovarian cancers. Black arrows indicate natural progression or activation pathways, while green arrows represent inhibitory effects of specific agents, such as the NF-κB inhibitor IKK-IV

Collectively, these findings weave a cohesive narrative: HDACs suppress antitumor immunity in gynecological cancers by altering the TIME, while HDACi counteract this by enhancing antigen presentation, invigorating CD8 + T-cell responses, and destabilizing immunosuppressive networks (Fig. 1). From cervical cancer’s synergy with bortezomib and vaccines to ovarian cancer’s modulation of Tregs, MDSCs, and CAR T-cell targets, HDACi stand as a cornerstone in immunotherapy, bridging epigenetic regulation with immune activation. Future research should focus on optimizing timing, dosing, and combinatorial regimens to fully harness their potential, ensuring they complement existing therapies like checkpoint blockade and adoptive cell therapies to transform outcomes in these challenging malignancies.

### Combining HDACi and DNMTi

The synergy of DNMTi and HDACi in combination therapy with immunotherapy lies in their complementary mechanisms that simultaneously enhance immune activation, dismantle immunosuppressive barriers, and optimize the tumor’s susceptibility to immune checkpoint blockade [149]. In immunocompetent mouse models of ovarian cancer, the triple combination of azacitidine, givinostat, and anti-PD-1 extended median survival by An impressive 14.5 days compared to mock-treated controls, outperforming standard chemotherapy such as paclitaxel in a rapidly progressing model. This regimen significantly enhanced the activation of critical immune subsets, including T and NK cells, and sensitized tumors to anti-PD-1 therapy—an effect not consistently observed with other HDACi like entinostat, suggesting that givinostat’s unique profile may better complement azacitidine’s immune-priming effects. Mechanistically, the combination leverages azacitidine’s ability to upregulate immune signaling pathways, such as IFN and chemokine production, while HDACi counteracts the potential upregulation of immunosuppressive PD-L1 that can accompany DNMTi-induced IFN signaling. This balanced modulation creates a TIME that is both highly immunogenic and less resistant to checkpoint inhibition [20].

Further evidence of synergy comes from studies combining azacitidine with Nexturastat A, an HDAC6i, which revealed additive reductions in PD-L1 and DNMT1 expression across multiple ovarian cancer cell lines. This dual targeting not only enhances immune recognition by lowering immunosuppressive signals but also reinforces epigenetic reprogramming to sustain antitumor immunity. For instance, the unexpected reduction of DNMT1 by Nexturastat A suggests a broader epigenetic crosstalk that amplifies azacitidine’s effects, potentially through shared pathways like p53 signaling, which regulates IFN-stimulated genes and pro-apoptotic responses. Additionally, the combination increased CXCL10 levels in both cell culture and ascites fluid, promoting immune cell recruitment and correlating with reduced tumor burden in models like ID8-VEGF-Defensin, which mirrors human ovarian cancer dynamics. These findings underscore the potential for DNMTi and HDAC6i to reshape the TIME in a way that maximizes the efficacy of anti-PD-1 therapy, creating a robust and sustained immune response [21]. The interplay between DNMTi and HDACi also involves intricate signaling networks, such as p53 and IFN pathways, which may be activated by DNA damage or immune stimulation, further enhancing immune cell recruitment and tumor cell death. While prolonged IFN signaling could risk inducing resistance to checkpoint inhibitors, the strategic dosing and timing of DNMTi and HDACi appear to mitigate this concern, optimizing immune activation without exhausting effector cells. These preclinical successes have catalyzed clinical exploration, including a phase II trial combining oral azacitidine (CC-486) with pembrolizumab in platinum-resistant ovarian cancer, with plans for future studies incorporating HDACi to enhance outcomes further [150]

**Fig. 2:**
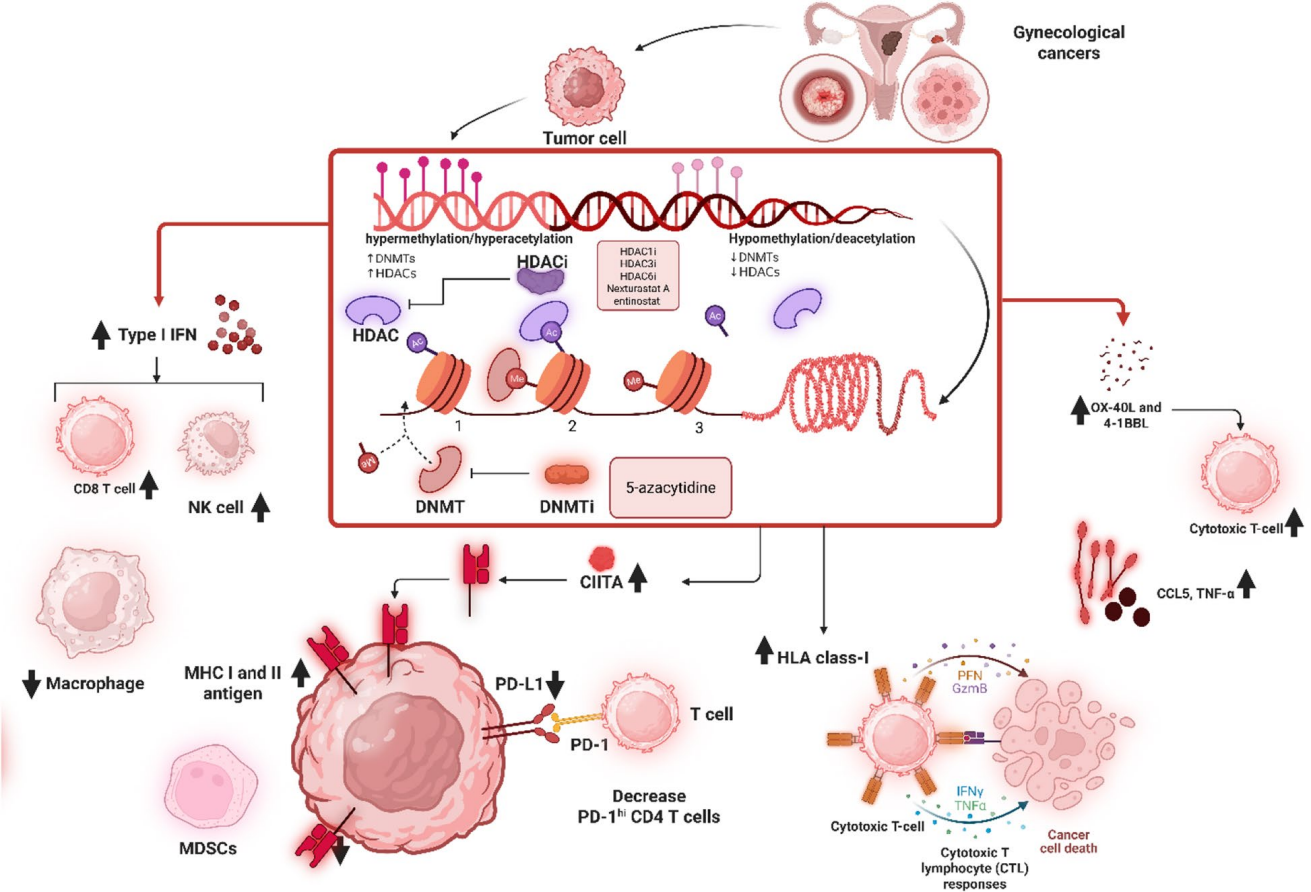
Epigenetic modulation of the TIME in gynecological cancers via EHMT inhibition. Tumor cells in gynecological malignancies leverage EHMT1/2-mediated H3K9me2 deposition to repress immune-stimulatory genes, including CXCL9 and CXCL10, facilitating immune evasion through chromatin compaction and gene silencing. EHMTi, using agents such as HKMTI-1–005 and EZM8266, reverses this suppression, enhancing IFNγ-mediated chemokine expression. This leads to increased CXCR3 + immune cell infiltration—including dendritic cells, NK cells, and cytotoxic CD8⁺ T cells—while reducing immunosuppressive CD4⁺ Tregs. Concurrently, EHMTi alleviates promoter hypermethylation and augments transposable element expression, thereby triggering antiviral mimicry pathways and amplifying interferon responses

As illustrated in Fig. 2, By orchestrating a dynamic interplay between tumor-intrinsic and immune-mediated effects, the combination of DNMTi and HDACi represents a transformative approach in immunotherapy for gynecological cancers. This strategy not only primes the TIME for effective checkpoint blockade but also holds promise for overcoming resistance mechanisms, paving the way for improved clinical responses. Continued research aims to refine these combinations, ensuring their translation into meaningful therapeutic advances for patients.

### EHMT and EHMT inhibitor

Euchromatin histone methyltransferase (EHMT), encompassing EHMT1 and EHMT2, is a critical epigenetic regulator responsible for depositing the repressive histone mark H3K9me2, which silences gene expression by compacting chromatin [157]. In the context of gynecological cancers, particularly ovarian cancer, EHMT plays a dual role; it contributes to tumor progression by epigenetically suppressing immunostimulatory genes, such as chemokines critical for immune cell recruitment, and it fosters therapy resistance, notably to Poly (ADP-ribose) polymerase inhibitors (PARPi). This silencing mechanism allows tumors to evade anti-tumor immune responses, creating an immunosuppressive TIME. However, targeting EHMT with inhibitors has emerged as a promising strategy to reverse this suppression, reactivating immune signaling pathways and enhancing the efficacy of cancer immunotherapy. The role of EHMTi in immunotherapy is particularly significant, as it not only derepresses key immune-related genes but also amplifies cytotoxic T-cell activity, offering a potential synergy with existing immunotherapeutic approaches [158, 159].

Research utilizing the ID8 Trp53-/- murine ovarian cancer model has demonstrated that a novel dual EHMT2/EZH2 inhibitor, HKMTI-1–005, significantly upregulates chemokine expression, including cxcl9, cxcl10, and ccl5, following IFNγ stimulation in vitro. In vivo, this inhibitor extended survival, reduced ascites accumulation, and shifted the TIME toward an immunostimulatory state, marked by increased infiltration of effector CD8 + T cells, NK cells, and DCs, alongside a reduction in immunosuppressive Tregs. These findings suggest that EHMT inhibition disrupts the tumor’s ability to maintain an immune-evasive niche, paving the way for enhanced immune recognition and attack. This immune remodeling links directly to the previous discussion of EHMT’s repressive role, illustrating how its inhibition can transform the TIME into a more favorable landscape for immunotherapy [160].

Expanding on this, studies of PARPi-resistant ovarian cancer models further highlight EHMT’s involvement in therapy resistance and its therapeutic potential when inhibited. Elevated EHMT1/2 expression and H3K9me2 levels in PARPi-resistant cells associate with suppressed transposable element (TE) expression, which, when derepressed by EHMTi, triggers a viral mimicry response. This response, mediated by dsRNA sensors RIGI and MDA5, activates interferon signaling pathways, boosting the expression of T-cell-recruiting cytokines and reducing tumor burden in immunocompetent models. Notably, in vivo experiments with PARPi-resistant ID8 TP53-/-/BRCA2-/- syngeneic models confirmed that both single EHMTi and combined EHMTi/PARPi treatments significantly reduced tumor growth, with effects partially dependent on CD8 + T cells. This dependency underscores a critical connection between EHMT inhibition and immune activation, building on the earlier evidence of chemokine-driven immune cell recruitment [161]. Further investigations using patient-derived ex vivo ovarian tumor cultures reinforce these findings, showing that dual EHMTi/PARPi therapy upregulates TE transcripts and immune signaling pathways, enhancing T-cell activity within the TIME, as evidenced by increased Granzyme B expression, a marker of cytotoxic T-cell function. Multispectral immunohistochemistry analyses revealed a remodeled immune microenvironment with heightened CD8 + T-cell activity, though recruitment dynamics remain less clear. This suggests that the role of EHMTi in immunotherapy extends beyond mere gene derepression to actively reshaping the TIME, potentially sensitizing resistant tumors to immune checkpoint blockade or other immunotherapies. The consistency of these effects across therapy-naïve and resistant models links back to the broader implications of EHMT’s epigenetic control, indicating its relevance across diverse disease states [162].

The mechanistic insights into EHMTi’s action further elucidate its immunotherapeutic promise. EHMT inhibition not only reduces H3K9me2 but also alters chromatin accessibility at TE loci, directly binding EHMT2 to these regions and promoting an open chromatin state that favors immune gene expression. In PARPi-resistant contexts, the interplay between EHMTi-induced TE reactivation and PARPi-driven DNA damage amplifies interferon responses via distinct pathways—MDA5 sensing TE-derived dsRNA and RIGI detecting PARPi-induced DNA:RNA hybrids. This dual activation enhances the immunostimulatory milieu, as seen in reduced tumor proliferation (via Ki67 staining) and increased cytokine production (e.g., CCL5, CXCL10, TNFα) in syngeneic models. These molecular shifts tie into the observed immune cell dynamics, offering a cohesive narrative of how EHMTi bridges epigenetic regulation with immune effector functions [163]. Importantly, EHMTi exhibits tumor-specific effects with low toxicity, as demonstrated by stable hematological parameters and confined EHMT1/2 expression to tumor cells in ovarian cancer models. This specificity, contrasted with the broader toxicity of other epigenetic therapies like DNA methyltransferase inhibitors, positions EHMTi as a viable candidate for clinical translation in gynecological cancers. Given that EHMT1/2 overexpression is a feature of various malignancies beyond ovarian cancer, including breast and pancreatic cancers, the therapeutic scope of EHMTi may extend to other gynecological and non-gynecological tumors [164].

**Fig. 3:**
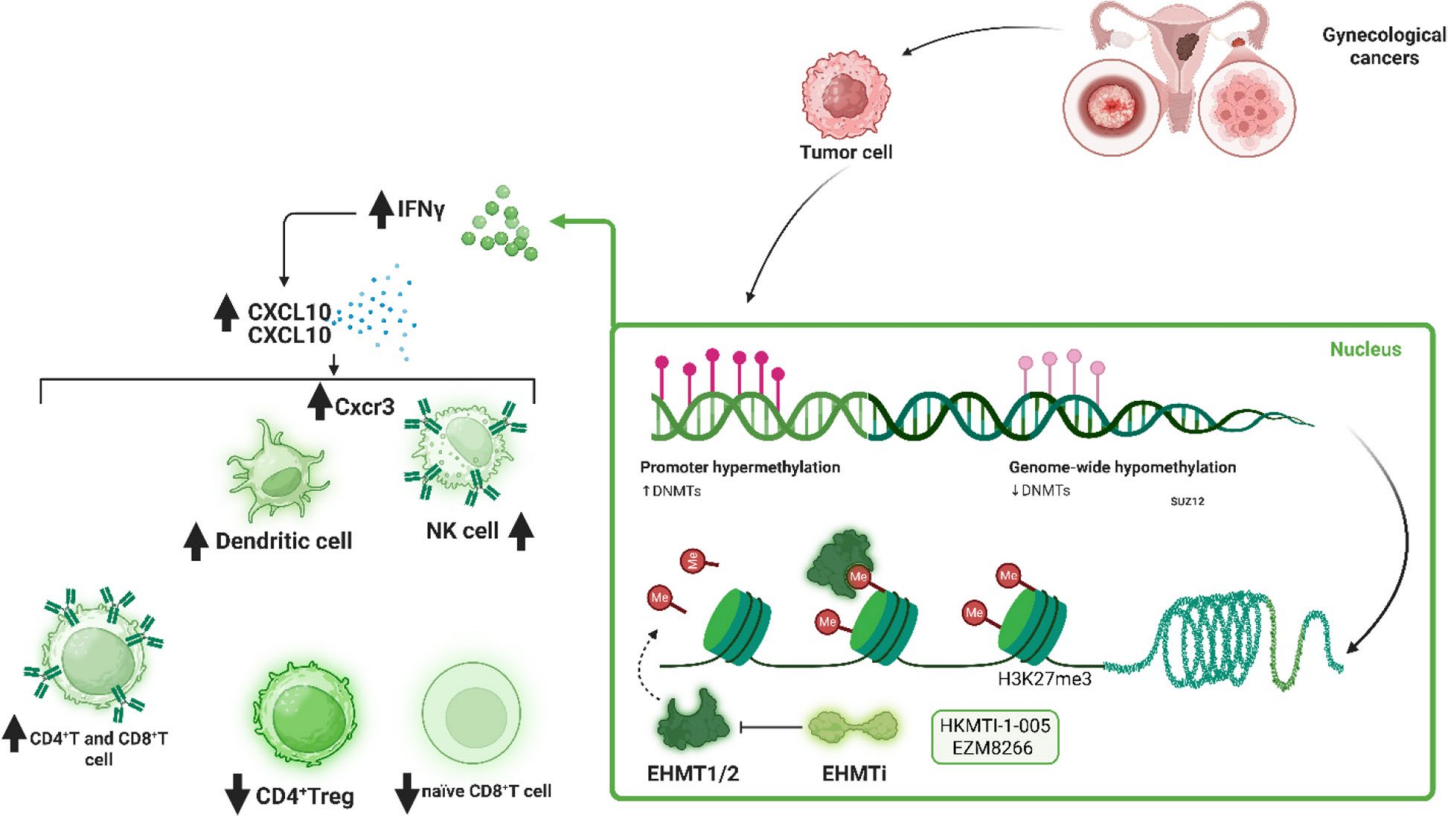
Mechanistic overview of BETi in modulating the TIME in gynecological cancers. BET proteins (BRD2, BRD3, BRD4, BRDT) are epigenetic readers that bind acetylated histones to regulate gene transcription. BET inhibitors such as i-BET151, ABBV-075, and JQ1 disrupt this interaction, leading to enhanced histone acetylation and altered gene expression. In ovarian and other gynecological cancers, BETi exert antitumor effects by promoting tumor cell apoptosis and reducing immune-suppressive CCR2⁺/CD68⁺ macrophages. They reprogram macrophages from an M2-like (protumor) to M1-like (antitumor) phenotype, disrupt PD-L1 expression, and inhibit immune escape. These changes collectively enhance CD8⁺ T cell infiltration, cytotoxic function, and immunogenic cancer cell death, offering a promising strategy to sensitize “cold” tumors to immunotherapy

To sum up, EHMT governs an epigenetic landscape that suppresses anti-tumor immunity in gynecological cancers, particularly through silencing chemokines and TEs critical for immune engagement. Its inhibition with EHMTi reverses this suppression, reactivating immune signaling, reducing tumor burden, and enhancing CD8 + T-cell-dependent responses in both PARPi-sensitive and resistant settings (Fig. 3). The consistent remodeling of the TIME across cellular, murine, and human ex vivo models underscores EHMTi’s potential as a versatile immunotherapeutic adjunct, offering a promising avenue to improve outcomes in gynecological cancers and beyond.

### BET and BET inhibitors

The bromodomain and extraterminal (BET) protein family, comprising BRD2, BRD3, BRD4, and the testis-specific BRDT, is an epigenetic regulators that bind acetylated lysine residues on histones and transcription factors to modulate gene expression [165]. These proteins are implicated in critical cellular processes such as apoptosis, cell cycle regulation, and cell invasion, making them attractive therapeutic targets [166]. In the context of ovarian cancer, as the most lethal gynecological cancer with the 5-year survival rate stagnates around 30%, BET inhibitors (BETi) have emerged as a promising strategy to enhance immunotherapy by reshaping the immunosuppressive TIME and boosting antitumor immunity [167, 168].

Studies have demonstrated that BETi, such as i-BET151 and JQ1, exert potent antitumor effects by targeting bromodomain-containing proteins [169]. In preclinical OC models, i-BET151 suppresses cancer cell viability by inducing apoptosis via the mitochondrial pathway while impairing invasion and migration. Notably, it reduces abdominal and lung metastases by diminishing the activity of immunosuppressive cells, including TAMs, and enhancing antitumor immune responses. Similarly, JQ1, the first developed BETi, inhibits the growth of NUT midline carcinomas driven by BRD4-NUT fusions and shows efficacy in MYC-driven cancers like neuroblastoma and melanoma. This dual action on tumor cells and the TIME underscores the role of BETi in immunotherapy, offering a novel approach to tackle ovarian cancer’s poor prognosis, which has seen little improvement despite advances in surgery and chemotherapy [170]. A key mechanism by which BETi influence the TIME involves reprogramming immunosuppressive TAMs, a hallmark of ovarian cancer’s “cold” tumor status characterized by limited cytotoxic T-cell infiltration. The CCL2/CCR2 axis, which recruits CCR2 + monocytes that differentiate into immunosuppressive TAMs, is a critical driver of resistance to therapies like anti-VEGF-A (AVA). Research shows that combining BETi ABBV-075 with AVA reduces TAM infiltration, shifts macrophages toward an M1-like antitumor phenotype, and inhibits M2-like protumor macrophages. A high M1/M2 ratio correlates with better prognosis and platinum sensitivity in ovarian cancer patients, while ABBV-075’s ability to downregulate CCR2 expression in macrophages disrupts the MSMP/CCR2-mediated adaptive resistance to AVA. This macrophage reprogramming complements BETi’s immunotherapeutic potential, enhancing cytotoxic T-cell responses and overcoming resistance without the toxicity limitations of CSF1R inhibitors, which can induce monocyte rebound effects [171].

Furthermore, BETi regulate immune checkpoint pathways, notably PD-L1 (encoded by CD274), a direct transcriptional target of BRD4 [172]. In ovarian cancer mouse models, JQ1 reduces PD-L1 expression on tumor cells, dendritic cells, and macrophages, relating with increased cytotoxic T-cell activity and slowed tumor progression. Given BRD4 amplification in OC and its correlation with CD274 expression, BETi provide a small-molecule alternative to anti-PD-L1 antibodies, which can cause immune-related adverse events with prolonged use. By suppressing both oncogenic and immune-associated PD-L1, BETi enhance immunotherapy with manageable toxicity, as evidenced by ongoing clinical trials. This broad regulation of TIME components—tumor cells, TAMs, Tregs, and PD-L1—positions BETi as a versatile tool to convert ovarian cancer’s immunologically unresponsive landscape into a more responsive state [173].

**Fig. 4:**
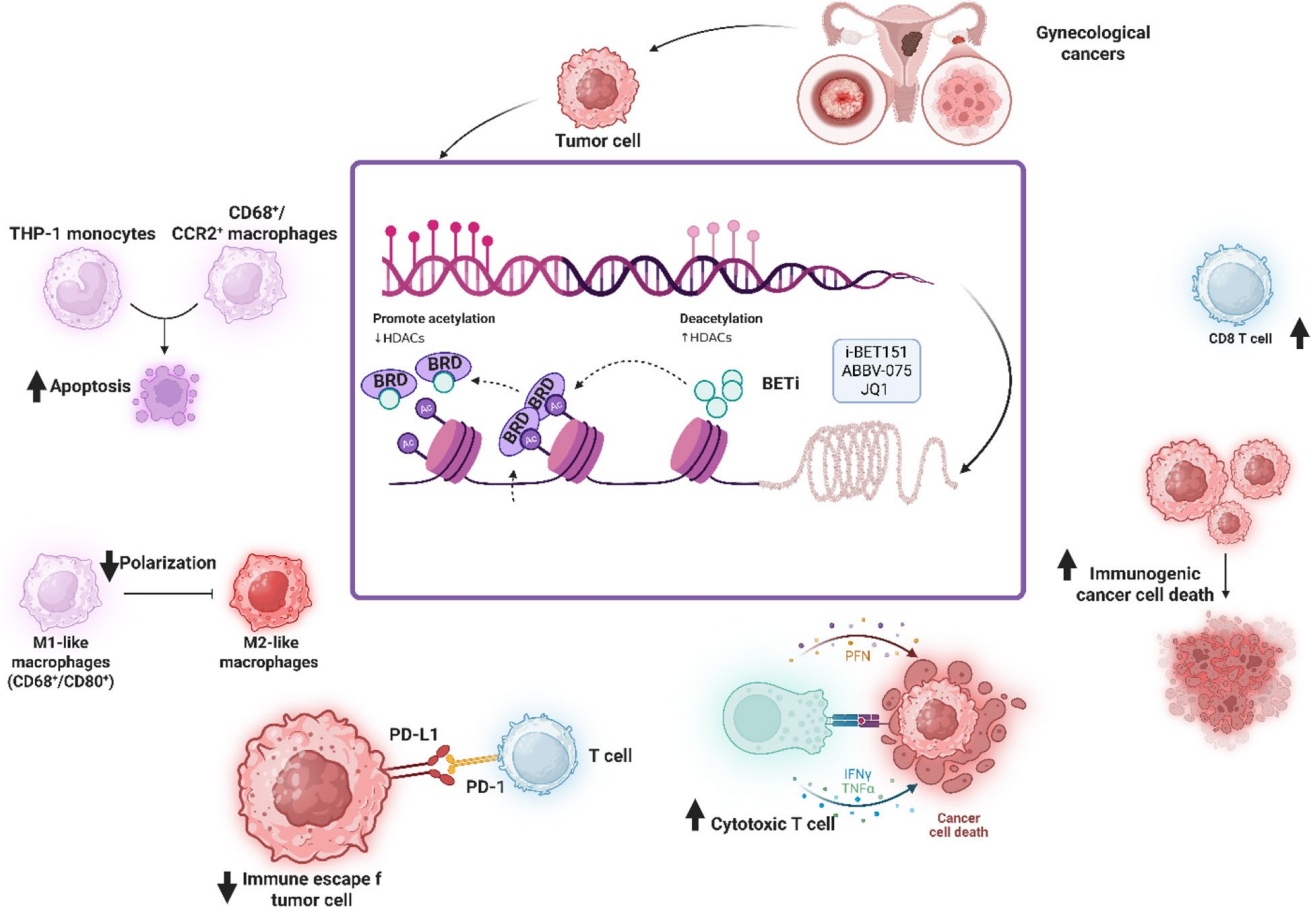
Mechanistic overview of EZH2-mediated epigenetic regulation and its immunotherapeutic implications in gynecological cancers. EZH2, a core component of the PRC2 complex, catalyzes H3K27me3 to silence tumor suppressor genes, such as TIMP2, thereby enhancing PD-L1 expression through NF-κB activation and promoting immune evasion. EZH2 also modulates promoter methylation dynamics, influencing chemokine expression like CCL22 and CXCL10, which shapes the immune microenvironment by recruiting immunosuppressive cells, Tregs and M2 macrophages, or enhancing cytotoxic lymphocyte infiltration. Inhibition of EZH2 and co-regulators like USP7 or G9A reverses these effects—restoring immune surveillance, reducing TAM polarization, and enhancing CAR-T cell efficacy. This positions EZH2 inhibition as a dual-action strategy targeting both tumor-intrinsic growth and immune evasion pathways in gynecological malignancies

In summary, BETi’s role in immunotherapy of gynecological cancers, particularly ovarian cancer, lies in their multifaceted ability to target epigenetic regulation and the TIME. From inducing tumor cell apoptosis and reducing metastases to reprogramming macrophages, modulating Tregs, and suppressing PD-L1, BETi like i-BET151, ABBV-075, and JQ1 offer a comprehensive strategy to enhance antitumor immunity (Fig. 4). Linking these effects, BETi bridge direct tumor suppression with immune activation, providing a foundation for combination therapies with AVA, vaccines, or checkpoint inhibitors. As clinical trials progress, further exploration of BETi’s immune-regulatory mechanisms and biomarkers like BRD4 expression will refine their therapeutic utility, potentially improving the dismal survival rates of ovarian cancer patients.

### EZH2i: an inhibitor of histone methyltransferase

Epigenetic regulation plays a pivotal role in the carcinogenesis of gynecological cancers, with Enhancer of zeste homolog 2 (EZH2) emerging as a critical epigenetic modulator. As the catalytic subunit of Polycomb Repressive Complex 2 (PRC2), EZH2 mediates H3K27me3, silencing tumor suppressor genes while promoting cell proliferation, migration, and immune evasion [174]. In cervical cancer, EZH2 overexpression drives aggressive phenotypes by epigenetically silencing TIMP2, a key inhibitor of matrix metalloproteinases, through promoter hypermethylation. This suppression disrupts extracellular matrix homeostasis and activates the NF-κB pathway, leading to upregulation of immune checkpoint molecules like PD-L1 and pro-tumorigenic cytokines comprising of TGF-β, VEGF, IL-6, and COX-2. Consequently, the TIME becomes immunosuppressive, facilitating immune escape. Notably, genetic or pharmacological inhibition of EZH2—or its stabilizer USP7—reverses these effects, restoring TIMP2 expression, dampening NF-κB signaling, and reducing PD-L1 levels. This mechanistic link positions EZH2 as a master regulator of immune evasion in cervical cancer, highlighting its therapeutic potential when targeted alongside immunotherapies [175]. Furthermore, EZH2-mediated epigenetic silencing of the CCL22-CCR4 axis in cervical cancer exemplifies its role in promoting metastasis and immune dysregulation. EZH2 suppresses DNMT3A, leading to hypomethylation of CCL22 and CCR4 promoters and subsequent overexpression of these genes, which recruit immunosuppressive Th2 cells and polarize macrophages toward an M2 phenotype. Targeting EZH2 or the CCL22-CCR4 axis reverses these effects, attenuating EMT and metastasis. Thus, EZH2 inhibitors not only disrupt tumor-intrinsic pathways but also restore immune homeostasis, offering a multifaceted approach to combat gynecological cancers [176, 177].

The role of EZH2 in modulating the TIME extends to ovarian cancer, where its inhibition synergizes with other epigenetic therapies to reshape immune landscapes. Dual blockade of EZH2 and the histone methyltransferase G9A (using inhibitors like HKMTI-1–005) has been shown to reprogram the TIME by increasing cytotoxic lymphocytes and NK cells while depleting immunosuppressive Tregs and TAMs. This combinatorial approach also impedes monocyte-to-macrophage differentiation and downregulates MARCO, a macrophage receptor linked to pro-tumor polarization. Transcriptomic analyses reveal that dual EZH2/G9A inhibition activates endogenous retroviruses (ERVs) and chemokine networks like as CXCL10, fostering a more immunogenic milieu. These findings underscore the dual utility of EZH2 inhibitors: they not only suppress tumor-intrinsic proliferation but also remodel the TIME to favor immune-mediated clearance, offering a strategic advantage in immunotherapy-resistant ovarian cancer [178]. Beyond intrinsic epigenetic modulation, EZH2 inhibitors significantly enhance the efficacy of adoptive T cell therapies, such as CAR-T and TCR-T cells, in gynecological cancers. Preclinical studies demonstrate that EZH2 inhibitors, tazemetostat and valemetostat, upregulate antigen HLA class I/II presentation machinery, OX40L, CD80 co-stimulatory ligands, and T cell-attracting chemokines, CXCL9 and CXCL10 in tumor cells. These changes improve CAR-T cell trafficking, synapse formation, and cytotoxic activity, overcoming common barriers in solid tumors. Notably, EZH2 inhibition does not compromise CAR-T cell fitness; instead, it preserves stem-like memory subsets (marked by TCF7/LEF1) and reduces PD-1, LAG3 exhaustion markers, promoting durable antitumor responses. This synergy between EZH2-targeted epigenetics and adoptive immunotherapy provides a compelling rationale for clinical trials in gynecological malignancies, particularly in PD-L1-high or immunologically"cold"tumors [179].

**Fig. 5:**
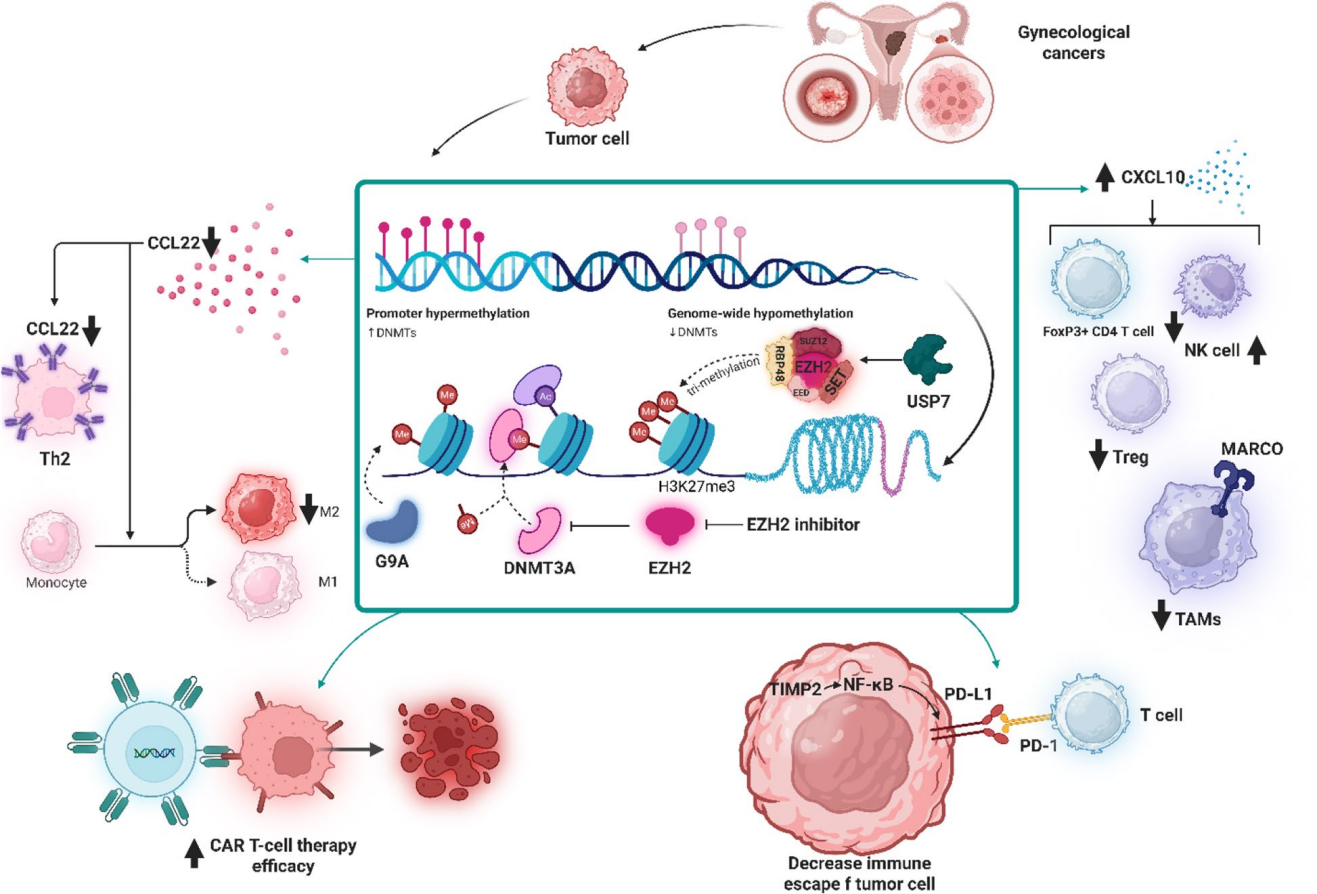
Mechanistic overview of EZH2-mediated epigenetic regulation and its immunotherapeutic implications in gynecological cancers. EZH2, a core component of the PRC2 complex, catalyzes H3K27me3 to silence tumor suppressor genes, such as TIMP2, thereby enhancing PD-L1 expression through NF-κB activation and promoting immune evasion. EZH2 also modulates promoter methylation dynamics, influencing chemokine expression like CCL22 and CXCL10, which shapes the immune microenvironment by recruiting immunosuppressive cells, Tregs and M2 macrophages, or enhancing cytotoxic lymphocyte infiltration. Inhibition of EZH2 and co-regulators like USP7 or G9A reverses these effects—restoring immune surveillance, reducing TAM polarization, and enhancing CAR-T cell efficacy. This positions EZH2 inhibition as a dual-action strategy targeting both tumor-intrinsic growth and immune evasion pathways in gynecological malignancies

In summary, EZH2 inhibitors hold transformative potential in gynecological cancer therapy by reshaping the TIME and augmenting immunotherapy. As shown in Fig. 5 From suppressing immune checkpoints like PD-L1 to enhancing CAR-T cell efficacy and disrupting metastatic pathways, EZH2 targeting represents a paradigm shift in epigenetic immunotherapy. Future research should focus on optimizing combinatorial regimens and translating these preclinical insights into clinical trials to improve outcomes for patients with gynecological cancers.

### LSD1

Lysine-specific histone demethylase 1 (LSD1/KDM1A), the first histone demethylase identified, plays a pivotal role in the epigenetic regulation of gene expression and is frequently dysregulated in gynecological cancers such as cervical and ovarian cancers [180]. By selectively removing methyl groups from mono- or di-methylated histone H3 lysine 4 (H3K4me1/2) or lysine 9 (H3K9me1/2), LSD1 can either repress or activate target genes, influencing tumor progression. Beyond histones, LSD1 demethylates non-histone substrates like p53, E2F1, and DNMT1, promoting aggressive cancer traits [181]. In cervical cancer, high LSD1 expression correlates with malignant progression, while its inhibition suppresses tumor growth and enhances immunotherapy efficacy, as observed in other cancers like melanoma and breast cancer. Recent evidence suggests that LSD1 ablation induces anti-tumor immunity by upregulating ERVs and disrupting the RNA-induced silencing complex, which leads to dsRNA accumulation and IFN β-dependent immune responses. This heightened immunogenicity, coupled with increased T-cell infiltration, overcomes resistance to immune checkpoint blockade, highlighting LSD1’s critical role in shaping the TIME and immunotherapy outcomes [182, 183].

LSD1 exerts direct and indirect regulatory effects on immune checkpoints, notably CD47 and PD-L1, which mediate tumor immune escape via the SIRPα/CD47 and PD-1/PD-L1 pathways, respectively [184]. Studies in cervical cancer demonstrate that LSD1 knockdown reduces CD47 and PD-L1 protein expression, with ChIP-Seq analyses revealing elevated H3K4me2 levels at the CD47 and PD-L1 promoter regions following LSD1 inhibition, indicating direct epigenetic regulation. Additionally, LSD1 modulates miR-34a, a microRNA that targets the 3′UTR of CD47 and PD-L1, through interactions with wild-type p53. LSD1 forms a stable complex with p53, and its inhibition increases the expression of p53 and miR-34a, thereby suppressing CD47 and PD-L1. In vivo, combining LSD1 inhibitors with anti-CD47/PD-L1 antibodies in TC-1 tumor-bearing mice significantly inhibits tumor growth by enhancing immune activation and reducing CD47 and PD-L1 signaling. These findings underscore LSD1’s dual regulatory axes—LSD1-H3K4me2-CD47/PD-L1 and LSD1-p53-miR-34a-CD47/PD-L1—as key mechanisms driving immune evasion in cervical cancer, positioning LSD1 inhibition as a promising strategy to augment immunotherapy [185].

In ovarian cancer, moderate overexpression of LSD1, particularly in stage IIIC and high-grade tumors, is linked to aggressive tumor signatures, including upregulated cell cycle genes and downregulated immune response genes, which correlate with poorer survival in some cohorts [186]. LSD1’s robust expression in ovarian cancer cell lines suggests a functional role, potentially independent of expression levels, similar to its necessity in hormonal signaling in breast and prostate cancers. The cytotoxic effects of LSD1 inhibitors in these cell lines further support its therapeutic potential, although in vivo validation is needed. Importantly, LSD1’s interaction with the SWI/SNF chromatin remodeling complex, observed in cancers like glioma, extends to gynecological malignancies. In small cell carcinoma of the ovary, hypercalcemic type (SCCOHT), LSD1 collaborates with SWI/SNF components to regulate gene expression. Inhibiting LSD1 with SP-2577 (Seclidemstat) induces ERV and IFNβ expression, promoting chemokine and PD-L1 upregulation, which enhances T-cell infiltration and tumor immunogenicity. This interplay between LSD1 and SWI/SNF highlights a broader epigenetic network influencing the TIME. [187].

Acetylation plays a critical role in immunotherapy by modulating chromatin accessibility and gene expression within the TIME. While LSD1 primarily regulates methylation, its inhibition indirectly influences acetylation dynamics through interactions with HDACs and SWI/SNF complexes, which recruit acetyltransferases [188]. In SCCOHT, LSD1 inhibition disrupts SWI/SNF-dependent ERV silencing, typically mediated by SMARCA4 or SMARCAD1, leading to increased histone acetylation at ERV loci and enhanced transcription. This acetylation-driven ERV activation generates tumor-specific antigens, amplifying T-cell responses. Furthermore, combining SP-2577 with anti-PD-L1 or anti-CTLA-4 antibodies in SCCOHT and OCCC organoids significantly boosts CD8 + T-cell infiltration, driven by acetylation-mediated upregulation of chemokines and immune checkpoints. The synergy between LSD1 inhibition and acetylation-enhancing therapies thus amplifies immunotherapy efficacy, sensitizing tumors to T-cell-mediated destruction [189].

LSD1’s multifaceted role in gynecological cancers bridges epigenetic regulation and immune modulation. Its inhibition not only disrupts tumor-promoting methylation but also enhances acetylation-dependent immunogenicity, reshaping the TIME to favor immune surveillance. By targeting LSD1, particularly in combination with checkpoint inhibitors, therapeutic strategies can exploit these epigenetic-immune axes to improve outcomes in cervical and ovarian cancers. The interplay between LSD1, SWI/SNF, and acetylation underscores a promising frontier for precision immunotherapy, warranting further exploration in clinical settings.

## Therapeutic approach

### Epidrugs and ICIs

Immunotherapy approaches that target the PD-L1/PD-1 pathway have shown impressive results in treating various cancers. However, due to the diverse nature of tumors and individual immune responses, PD-L1/PD-1 inhibitors often yield slow response rates in many patients [190]. Research highlights that a successful response to these therapies depends on maintaining a cohesive immune cycle. Disruptions at any stage of this cycle are a primary reason for treatment failure. Such disruptions can be addressed through epigenetic modifications, which can reshape the tumor's immune environment, enhance tumor antigen presentation to trigger immune responses, and improve T cell movement and reactivation [191]. Combining PD-L1/PD-1 inhibitors with epigenetic therapies holds significant promise for retraining the immune system and enhancing the effectiveness of checkpoint inhibitor treatments. For example, the DNA demethylating agent decitabine, when paired with anti-PD-1, bolstered CD8 + progenitor T cell proliferation and effector function, sustaining antitumor activity through epigenetic remodeling [192]. These findings underscore the potential of epidrugs in enhancing ICI therapy by reprogramming the TME and invigorating immune responses, setting a precedent for their application in gynecological cancers.

In gynecological cancers, particularly ovarian HGSC, epidrugs such as azacitidine and HDAC inhibitors play a pivotal role in ICI therapy by counteracting the immunosuppressive TME [193]. Studies using the ID8 mouse ovarian cancer model revealed that ex vivo azacitidine treatment of tumor cells significantly increased immune cell recruitment to the ascites, reduced tumor burden, and heightened sensitivity to anti-PD-1 therapy. In vivo experiments further demonstrated that combining azacitidine with HDAC inhibitors, Entinostat or Givinostat, and anti-PD-1 resulted in the most substantial reduction in tumor burden, prolonged survival, and increased activated T and NK cell populations, while decreasing MDSCs [194]. Transcriptomic analyses from the TRIO026 phase II clinical trial (NCT02900560) corroborated these findings, showing that azacitidine and pembrolizumab upregulated inflammatory and cytolytic genes GZMA and GZMH and enriched pathways like IFNγ response and NK cell-mediated cytotoxicity in platinum-resistant ovarian cancer. These changes were associated with a reshaped TME, marked by greater intra-tumoral T cell density and prolonged treatment duration, highlighting epidrugs’ ability to amplify ICI efficacy through immune activation [195, 196].

The role of specific HDAC inhibitors, such as ACY1215, an HDAC6 inhibitor, further illustrates epidrugs’ therapeutic potential in ICI therapy for gynecological cancers with ARID1A mutations [197]. ACY1215 activates CD4 + and CD8 + T cells, increases IFNγ-producing T cells, and suppresses ARID1A-mutated tumors in immunocompromised models [198]. Notably, ARID1A mutations, prevalent in > 50% of clear cell ovarian cancers, correlate with increased PD-L1 expression due to direct repression of CD274 gene transcription or heightened mutation loads. The combination of ACY1215 with anti-PD-L1 enhances antitumor immunity by reshaping the TME, with CD8 + T cell depletion abrogating these effects, underscoring the immune-mediated mechanism. Additionally, inactivation of the PBAF complex in ARID1A-mutated tumors increases sensitivity to IFNγ, promoting chemokine secretion and effector T cell recruitment [197]. These findings position epidrugs as critical enhancers of ICI therapy, particularly for ARID1A-mutated gynecological cancers, by leveraging epigenetic mechanisms to bolster immune responses.

Beyond anti-PD-1 therapy, epidrugs like decitabine demonstrate synergy with anti-CTLA-4 in epithelial ovarian cancer, as evidenced by orthotopic mouse models using the BR5FVB1-Akt cell line. Decitabine treatment upregulates immune-related genes, including CCL2, CCL5, CXCL10, and Tnfsf18 (GitrL), which enhance NK and CD8 + T cell infiltration And function. This leads to reduced tumor growth, delayed ascites formation, And fewer MDSCs, with 40% of treated mice achieving complete responses. The combination of decitabine and anti-CTLA-4 sustains a prolonged cytotoxic lymphocyte response, increasing IFNγ and TNFα production and promoting effector T cell differentiation. DAC also induces CD80 expression on cancer cells, amplifying tumor-specific cytotoxic T lymphocyte responses. These effects are distinct from azacitidine, as decitabine directly augments NK cell cytotoxicity without impairing function, further emphasizing epidrugs’ versatility in enhancing immunotherapy by modulating both innate and adaptive immunity [170].

In conclusion, epidrugs such as azacitidine, decitabine, and HDAC inhibitors significantly enhance ICI therapy in gynecological cancers by reprogramming the immunosuppressive TME, upregulating immune-related genes, and promoting effector T and NK cell infiltration and activity. These agents demonstrate particular promise in ARID1A-mutated tumors and platinum-resistant ovarian cancers, offering a robust strategy to overcome immunotherapy resistance (Table 3). While their role in CAR-T cell therapy requires further exploration, the immunomodulatory prowess of epidrugs positions them as a cornerstone of innovative immunotherapeutic approaches, paving the way for improved clinical outcomes in gynecological cancers.

**Table 3:** Epigenetic drug combinations and their immunomodulatory effects in ovarian cancer

Epigenetic drugs	Immunotherapy	Dosage	Combination immunotherapy	Immune Target of Epidrug	Cancer and cell types	Study Type	highlights	Ref
Nexturastat A 5-azacytidine	HDAC6i DNMTi	Varying levels 500 nM	ICI: anti-PD-1	↑Type I interferon response, ↑cytokine and chemokine expression, and ↑MHC I antigen presentation complex, ↑IFNg + CD8, NK, and NKT ↓MDSCs and PD-1^hi^ CD4 T cells	Ovarian A2780, Hey, Kuramochi, TykNu, SKOV3, IGROV-1 CR, SKOV3 CR	In vitro and in vivo	“Combining DNMTi and HDAC6i in ovarian cancer cell lines enhanced immunogenicity, increasing type I interferon signaling, cytokine production, and MHC I expression more than either treatment alone. However, in the ID8 MOSE Trp53 −/− mouse model, this combination only modestly improved survival while promoting a more immunogenic tumor microenvironment Adding anti-PD-1 therapy did not improve outcomes, likely due to the extremely high PD-L1 levels induced by the DNMTi + HDAC6i combination, which may have hindered the efficacy of immune checkpoint blockade.”	[21]
Azacitidine	DNMTi		ICI: anti-CTLA4 and anti-PD-1	↑β2 microglobulin (β2m), CTLA4, PD-1, PD-L1	High-grade serous ovarian cancer (HGSOC) KURAMOCHI, OVSAHO, COV362, OVCAR4, COV318, TYKNU, OVKATE, and OAW28	In vitro	“Sequential azacitidine and platinum-based chemotherapy on HGSOC can increase expression of transcripts associated with immune responses, whilst occurring in parallel to decreased proliferation and/or increased cell death, therefore priming platinum-resistant HGSOC for increased response to CPI immunotherapy.”	[215]
5-azacytidine Givinostat	DNMTi HDACi		ICI: anti-PD-1	↑T and NK cells, ↓macrophages	Ovarian MOSEC, Roby-ID8-luc2, Roby-ID8-nonluc, and ID8-VEGF-defensin	In vitro and in vivo	“Combining 5-azacytidine and givinostat enhances ovarian cancer response to immune checkpoint blockade by modulating both tumor cells, inducing immune gene upregulation, apoptosis, and interferon signaling, and immune cells, activating T/NK cells and reducing macrophages in the tumor microenvironment, ultimately reducing tumor burden and improving survival in immunocompetent mouse models.”	[216]
UNC0642, EZM8266	Euchromatic histone lysine methyltransferases 1/2 inhibitor(EHMT1/2i)	1 μmol/L		↑CD8 T-cell	Ovarian PEO1, Kuramochi, OVCA420, and HGS2	In vitro and in vivo	“Combinatory EHMT and PARP inhibition stimulates a cell-autologous immune response in vitro, is an effective therapy for reducing PARPi-resistant ovarian tumor growth in vivo, and promotes antitumor immunity activity in the tumor microenvironment of patient-derived ex vivo ovarian cancer tissues.”	[163]
Entinostat, Givinostat 5-azacytidine	HDAC6i DNMTi		ICI: anti-PD-1	↑T and NK cells ↓MDSCs	HGSC	In vitro and in vivo	“Tumor cells treated with AZA effectively recruit a higher number of immune cells to the tumor ascites. HDACis and anti-PD1 are less effective in this regard when administered as single agents; however, this effect is significantly enhanced when used in combination with a demethylating agent.”	[194]
5-azacytidine	DNMTi		ICI: Pembrolizumab (anti-PD-1)	↑IFNG, ↑CXCL13, ↑CXCR5, ↑CD8 T cell and NK cell	Ovarian	Phase II clinical trial	“The combination of azacitidine and pembrolizumab reshapes the tumor microenvironment by enhancing inflammatory and immune responses, potentially improving efficacy in platinum-resistant ovarian cancer”	[195]
Decitabine	DNMTi	1.0 mg/kg	ICI: anti-CTLA-4	↑NK cells, CD8^+^ T-cell, ↑IFNγ and TNFα	Ovarian BR5FVB1-Akt	In vitro and in vivo	“Low-dose decitabine treatment increases the expression of chemokines that recruit NK cells and CD8^+^ T cells, promotes their production of IFNγ and TNFα, and extends the survival of mice bearing subcutaneous or orthotopic tumors. The efficacy of anti–CTLA-4 was potentiated by combination with decitabine.”	[217]
Decitabine	DNMTi	1 μM daily	CAR-T cells	chondroitin sulfate proteoglycan 4 (CSPG4)	Ovarian SKOV-3, Caov-3	In vitro	“Decitabine-mediated upregulation of CSPG4 on SKOV-3 ovarian cancer cells enables antigen-specific targeting using CSPG4-CAR-T cells resulting in effective CSPG4-directed target cell killing even in the presence of Decitabine.”	[210]
Decitabine	DNMTi	0.015–32 μΜ		↑MHC-class I and ↑PD-L1, ↑IFN-related genes	Cervical CaSki, C33A	In vitro	“Decitabine treatment significantly reduces cell viability, induces G2/M phase cell cycle arrest and apoptosis and increases response to chemotherapy. Furthermore, the prolonged upregulation of MHC-class I and PD-L1 in CC cells has been demonstrated.”	[218]
Decitabine	DNMTi	1.25 mg/kg		CD3, CD4 and CD8 + T cells, IFN-γ and MIP-1β	Ovarian ID8-RFP	In vitro and in vivo	“ The outcome of combination treatment decitabine and selinexor was accompanied by an increase in granzyme B secretion in CD8 + T cells and by a significant release of all the macrophage and T cell cytokines.”	[219]
Entinostat	HDACi	5 μM	ICI: anti-PD-1	↑IFNγ^+^CD4^+^T cells, ↑TNFα^+^CD4^+^T cells, ↑TNFα^+^CD8^+^T cells, ↑IFNγ^+^NK cells, PRF1^+^NK cells, ↑M1 macrophages and CD86^+^ DC	Ovarian SKOV3, OVCAR5 and CAOV3	In vitro and in vivo	“Niraparib, a PARPi, combined with Entinostat induced HRD-EXCUTE by activating the cGAS-STING pathway and increasing the histone acetylation. The combination therapy could enhance the cytotoxicity of immune cells and promote pro-immune cells infiltrating into ascites, resulting in inhibited ovarian cancer growth.”	[220]
ACY1215	HDACi	50 mg/kg daily	ICI: anti-PD-L1	↑IFNγ^+^CD8 T	Ovarian clear cell carcinoma OVCA429	In vitro and in vivo	“Inhibition of HDAC6 synergizes with anti-PD-L1 immune checkpoint blockade in ovarian cancer with ARID1A inactivation. ARID1A directly repressed the transcription of *CD274*, the gene encoding PD-L1. A reduced tumor burden and improved survival were observed in ARID1Aflox/flox/PIK3CAH1047R ovarian clear cell carcinoma (OCCC) mice treated with the HDAC6 inhibitor ACY1215 and anti-PD-L1 immune checkpoint blockade, resulting from the activation and increased presence of interferon-gamma-positive CD8 T cells.”	[197]
Vorinostat	HDAC6i	10 nM		↓IL-10, ↓CD206, ↓M2 macrophage polarization	Endometrial IOSE, 12Z, TOV-21 G, TOV-112D	In vitro and in vivo	“ARID1A6488delG/HDAC6 induced M2 polarization of macrophages through the production of IL-10. Vorinostat inhibited cell growth and blocked the activity of HDAC6, significantly reducing the size of ovarian tumors by inhibiting the polarization of M2 macrophages in mice.”	[221]
Vorinostat	HDAC6i	10 μM		↑MHC Class I Polypeptide-Related Sequence A (MICA), ↑NK cell-mediated killing of cancer cells	Cervical SiHa and HeLa	In vitro and in vivo	“Vorinostat suppressed the proliferation, migration, and invasion of cervical cancer cells while inducing apoptosis and S-phase cell-cycle arrest, downregulating the PI3K/Akt signaling pathway, upregulating MICA expression both in vitro and in vivo, and enhancing NK-92 cell-mediated cytotoxicity by modulating the PI3K/Akt pathway.”	[222]
Vorinostat	HDAC6i	2.5 µmol/l	AA98: an anti-CD146 monoclonal antibody	↑CD146	Ovarian A2780, SKOV3 and Caov3	In vitro and in vivo	“Targeting CD146 substantially enhanced vorinostat-induced killing by suppressing the activation of Akt pathways in ovarian cancer cells. AA98, in combination with vorinostat, significantly inhibited cell proliferation and increased apoptosis. In vivo, AA98 synergized with vorinostat to inhibit tumor growth and prolong survival in ovarian cancer.”	[204]
Romidepsin	HDAC6i		CAR-T cells	↑IFN- γ, ↑IL-2, ↑killer cell lectin-like receptor subfamily K member 1 ligands (NKG2DL), ↑pore-forming protein (PFP)	Ovarian serous cystadenocarcinoma SKOV3	In vitro	“Upregulating the expression of antigens of ovarian cancer cells can enhance the cytotoxicity of CAR-T cells. Targeting NKG2DL by NKG2D-NKG2DL binding in CAR-T cell design is promising when combined with romidepsin.”	[212]
Panobinostat	HDACi	50 nM		T cell, CD274/PD-L1, TNFSF9 and IL-8, CXCL3	HGSOC SKOV-3	In vitro	“Olaparib And panobinostat treatment significantly reduced the expression of 20 genes, including BRCA1, BRCA2, and WEE1. Examples of other significantly enriched functional pathways were cytokine secretion, inflammatory response genes, T cell activation, and immune cell recruitment. Consistent with these RNA-seq data, the combined treatment significantly reduced ID8-luc tumor burden and cell growth, while increasing apoptosis and DNA damage.”	[223]

### Epidrugs and monoclonal antibodies

Epigenetic modulators, or epidrugs, have emerged as promising agents in enhancing the efficacy of monoclonal antibody (mAb) therapies across various cancers, including hematological malignancies [199]. For instance, HDACis such as SAHA, vorinostat, have demonstrated synergistic effects when combined with the anti-CD20 mAb rituximab in mantle cell lymphoma (MCL). This combination induces apoptosis through caspase activation and Bcl-2 downregulation, disrupting cytoprotective pathways like NF-κB, Akt, and ERK1/2, while activating pro-apoptotic JNK pathways. In vivo studies further confirmed that SAHA and rituximab significantly reduced tumor growth and prolonged survival in MCL-bearing mice without altering CD20 expression [200]. Similarly, DNMTis have been shown to upregulate CD38 expression on multiple myeloma (MM) plasma cells, enhancing the anti-MM efficacy of daratumumab via increased antibody-dependent cellular cytotoxicity [201]. These findings highlight the potential of epidrugs to amplify mAb-mediated immunotherapy, setting a precedent for their application in gynecological cancers.

In gynecological cancers, particularly ovarian cancer, HDACis like vorinostat have shown significant promise as second-line therapies due to their tumor-specific action [202]. However, their standalone potency is limited, often triggering both pro- and anti-apoptotic responses due to their broad molecular targets [203]. Research has identified that vorinostat induces the expression of CD146, an adhesion molecule, in ovarian cancer cells both in vitro and in vivo, which paradoxically acts as a protective mechanism, reducing the drug’s antitumor efficacy. To counter this, combining vorinostat with AA98, a CD146-targeting agent, has proven effective. This regimen enhances apoptosis, inhibits cell proliferation, and prevents colony formation in ovarian cancer cells. In vivo studies with SKOV3 xenografts demonstrated that vorinostat plus AA98 significantly reduced tumor volume and extended survival compared to single-agent treatments, underscoring the synergistic potential of targeting CD146 alongside HDACis. The efficacy of this combination is closely tied to the modulation of intracellular signaling pathways, particularly the Akt pathway, which mediates anti-apoptotic signals in cancer cells. Co-administration of AA98 with vorinostat attenuates Akt phosphorylation And downregulates 4E-BP1 expression, enhancing the sensitivity of ovarian cancer cells to vorinostat-induced apoptosis. Additionally, AA98 reverses vorinostat-induced activation of S6K1, further sensitizing cells to treatment. These findings align with observations in other cancers, such as cervical cancer, where Akt signaling influences HDACi sensitivity, though factors like S6K1 can independently modulate responses. Overexpression and inhibition experiments have confirmed Akt’s protective role in vorinostat/AA98-induced apoptosis, highlighting the critical interplay between epigenetic regulation and signaling pathways in optimizing HDACi-based therapies [204, 205].

The integration of epidrugs like HDACis with mAb therapies in gynecological cancers represents a novel therapeutic frontier. By targeting protective mechanisms such as CD146 induction and modulating key signaling pathways like Akt, these combinations can overcome drug resistance and enhance tumor cell killing. However, the development of optimal HDACi-based regimens requires rigorous clinical trials to refine dosing and combination strategies, as current approaches remain largely empirical. Preclinical studies, such as those combining vorinostat and AA98, provide a foundation for designing mechanism-based therapies that maximize the immunotherapeutic potential of epidrugs. Continued research into epigenetic-immunotherapy synergies will be crucial for improving outcomes in patients with advanced gynecological cancers (Table 4).

**Table 4:** Summary of clinical trials evaluating epigenetic therapies in gynecological cancers

Drug Class	Drug(s)	Cancer Type	Trial Phase	Status	Key Outcomes	PMID
HDAC Inhibitor	Belinostat	Ovarian (EOC, LMP)	II	Completed	PR: 1 (LMP, unconfirmed), SD: 10 (LMP), 9 (EOC), Median PFS: 13.4 months (LMP), 2.3 months (EOC)	20,304,628
HDAC Inhibitor	Vorinostat + Carboplatin + Paclitaxel	Ovarian (Platinum-Sensitive)	II	Completed	ORR: 62.2%, CR: 14, PR: 19, Median PFS: 11.6 months, Median OS: 40.6 months	38,337,591
HDAC Inhibitor	Belinostat + Carboplatin + Paclitaxel	Ovarian (EOC)	Ib/II	Completed	ORR: 43%, CR: 3, PR: 12, Median PFS: 48% at 6 months	22,694,911
DNA Hypomethylating Agent	Decitabine + Carboplatin	Ovarian (Partially Platinum-Sensitive)	II	Completed	ORR: 6/13 (combination) vs 9/14 (carboplatin alone), Median PFS: not reported	24,642,620
BET Inhibitor	RO6870810 + Atezolizumab	Ovarian, TNBC	Ib	Terminated	PR: 2 (5.6%), Limited antitumor activity, High immune-related AEs	40,102,759
Immune Checkpoint Inhibitor	Pembrolizumab	Endometrial (MSI-H)	II	Completed	ORR: 58%, 100% (Lynch-like) vs 44% (sporadic), Median PFS: 100% vs 30% at 3 years	33,932,502

### Epidrugs and CAR-T cells therapy

Epigenetic modifications play a pivotal role in regulating gene expression, and their dysregulation is a hallmark of many cancers, including gynecological malignancies. Epidrugs, such as DNMTis and HDACis, have emerged as powerful tools to enhance the efficacy of immunotherapies, particularly CAR-T cell therapy, by modulating the tumor microenvironment and immune cell function [206]. In cancers beyond gynecological Malignancies, epidrugs have shown promise in optimizing CAR T-cell therapy. For instance, in pediatric brain tumors, DNMTis Like 5-azacytidine and GSK-3484862 have been used during CAR T-cell manufacturing to counteract T-cell exhaustion, enhancing proliferation, persistence, and cytotoxicity against B7-H3-positive tumor cells. These epidrugs promote an enriched memory T-cell phenotype and remodel epigenetic exhaustion markers, leading to sustained anti-tumor activity [207]. Similarly, in multiple myeloma, the HDACi entinostat has been employed to stabilize CAR expression in CD138-targeting CAR-NK cells, improving their persistence and tumor-killing capacity in vivo [208]. Additionally, in high-grade chondrosarcoma, the HDACi vorinostat upregulates B7-H3 expression on tumor cells and enhances the cytotoxic activity of B7-H3-specific CAR T-cells, demonstrating the versatility of epidrugs in amplifying immunotherapy across various cancer types [209]. These findings establish a foundation for exploring epidrugs in gynecological cancers, where analogous epigenetic strategies are being studied to address the challenges of CAR T-cell therapy in solid tumors.

In gynecological cancers, particularly ovarian cancer, epidrugs such as decitabine And romidepsin have been instrumental in enhancing CAR T-cell therapy by upregulating target Antigens on tumor cells. Decitabine, a DNMTi, has been shown to induce the expression of chondroitin sulfate proteoglycan 4 (CSPG4) on ovarian cancer cells, such as SKOV-3, in a dose-dependent manner. By demethylating the CSPG4 promoter, decitabine converts over 50% of treated ovarian cancer cells into CSPG4-positive targets, enabling effective targeting by CSPG4-specific CAR T-cells. This approach marks the first demonstration of de novo CSPG4 upregulation followed by successful CAR T-cell-mediated cytotoxicity, highlighting decitabine’s role in creating inducible secondary antigens for immunotherapy. Importantly, the antigen-specific killing of decitabine-treated ovarian cancer cells is not impaired by the presence of the drug, and optimal dosing regimens (1 µM Daily for 5–8 days) have been established to maximize CSPG4 expression [210]. However, challenges remain, as CSPG4-negative tumor cells persist, necessitating combinational strategies. For example, dual-targeting approaches that pair CSPG4-specific CAR T-cells with those targeting other ovarian cancer antigens, such as ErbB2, mesothelin, or TAG-72, could address this limitation. Additionally, decitabine has been explored in conjunction with mRNA-electroporated CAR T-cells, which offer transient CAR expression to mitigate on-target/off-tumor toxicity, a critical concern given CSPG4’s low-level expression in normal tissues like the small intestine [211].

Similarly, romidepsin, an HDACi, has been utilized to enhance the expression of NKG2D ligands (NKG2DL), such as MICA/MICB, on ovarian cancer cells, thereby improving the efficacy of NKG2DL-targeting CAR T-cells. By increasing antigen density, romidepsin significantly enhances immune synapse formation, leading to near-saturation killing effects at high effector-to-target ratios. This approach not only boosts cytotoxicity but also allows for the use of fewer CAR T-cells, potentially reducing the risk of cytokine release syndrome, a major clinical challenge [212]. Unlike decitabine, romidepsin does not significantly alter cytokine secretion, suggesting a more targeted enhancement of cytotoxicity. Furthermore, romidepsin does not negatively impact the immunosuppressive tumor microenvironment, as evidenced by unchanged PD-1 secretion levels, making it a clinically safe option [213, 214]. Future strategies combining romidepsin with immune checkpoint inhibitors, such as anti-PD-1 or anti-PD-L1 antibodies, could further amplify CAR T-cell efficacy by simultaneously addressing antigen expression and immunosuppression. The synergy between epidrugs like decitabine and romidepsin underscores their potential to transform ovarian cancer immunotherapy by making tumor cells more recognizable to engineered T-cells.

Altogether, epidrugs like decitabine and romidepsin represent a transformative approach in gynecological CAR T-cell therapy by epigenetically reprogramming tumor cells to express target antigens, thereby enhancing the specificity and efficacy of engineered T-cells. Their ability to upregulate antigens such as CSPG4 and NKG2DL addresses the critical challenge of low antigen density in solid tumors, while their compatibility with combinational strategies offers hope for overcoming tumor heterogeneity (Table 3). Future research should focus on validating these findings in vivo, particularly through comprehensive screening of primary ovarian cancer cells for epidrugs’ inducibility and assessing off-tumor effects in normal tissues. Integrating epidrugs with immune checkpoint inhibitors and advanced CAR T-cell engineering platforms, such as mRNA-based or dual-targeting systems, holds promise for developing more effective and safer immunotherapies. By harnessing the power of epigenetic regulation, epidrugs pave the way for personalized and precise treatments for gynecological cancers, potentially improving outcomes for patients with limited therapeutic options.

## Clinical trials

Gynecological cancers, including ovarian and endometrial carcinomas, pose significant therapeutic challenges due to their resistance to conventional treatments like chemotherapy and radiation [3]. The critical role of epidrugs in gynecological cancers has been increasingly recognized in clinical trials, as these agents target epigenetic alterations that drive tumor progression and treatment resistance. Epidrugs, such as HDACi and DNMTi, modulate gene expression to restore sensitivity to therapies [224]. A phase II study of the HDACi belinostat in platinum-resistant EOC and low Malignant potential ovarian tumors demonstrated its tolerability And efficacy, with a median PFS of 13.4 months in low Malignant potential tumors And stable disease in 9 of 18 EOC patients. Evidence of histone acetylation in peripheral blood mononuclear cells and tumor tissue underscored belinostat’s epigenetic activity [225]. Similarly, a phase II trial combining the HDACi vorinostat with paclitaxel And carboplatin in platinum-sensitive recurrent ovarian cancer reported a 62.2% ORR And a median PFS of 11.6 months, highlighting epidrugs’ potential to enhance chemotherapy responses [226]. A phase 1b/2 study of belinostat with carboplatin and paclitaxel (BelCaP) in recurrent EOC achieved a 43% ORR, with a 48% 6-month PFS, particularly notable in heavily pretreated patients [227]. However, a trial combining the DNMTi decitabine with carboplatin in partially platinum-sensitive ovarian cancer was terminated early due to reduced efficacy And high toxicity, including 60% grade 3/4 neutropenia, indicating challenges in epidrug scheduling and delivery [228]. These trials collectively emphasize epidrugs’ transformative potential in gynecological cancers, while highlighting the need for optimized strategies to maximize their clinical impact.

When combined with immunotherapy, epidrugs aim to reprogram the tumor microenvironment to enhance immune recognition and overcome resistance to ICIs [229]. A phase II study (NCT02901899) investigated the hypomethylating agent guadecitabine with pembrolizumab in PROC. The trial reported a modest response rate (RR) of 6.6%, with 2 partial responses And a clinical benefit rate of 27%, including 16 patients with stable disease. Notably, guadecitabine induced significant LINE1 hypomethylation in peripheral blood mononuclear cells And differential methylation of 39,579 CpG sites in tumors, affecting pathways like endosomal transport. Increased antigen-specific cytotoxic T-cell activity in CD8 + cells from ascites suggested epidrug-mediated immune priming, though the limited RR indicated persistent immune suppression in PROC [72]. In contrast, a phase 1b study combining the BETi RO6870810 with atezolizumab in advanced ovarian cancer and triple-negative breast cancer was terminated early due to severe immune-related adverse events, including systemic immune activation. Despite confirmed BETi target engagement, RO6870810 failed to reduce tumor PD-L1 expression And suppressed Antitumor immunity, with only atezolizumab driving immune effector activation in the tumor microenvironment. The trial reported a 5.6% RR, with 2 PRs, underscoring the challenges of combining epidrugs with ICIs due to heightened toxicity without synergistic antitumor effects [230].

The complexity of epidrug-immunotherapy combinations is further highlighted by biomarker-driven studies. A phase II study (NCT02899793) of pembrolizumab in microsatellite instability-high (MSI-H) endometrial cancer demonstrated a 58% ORR, with 100% ORR in Lynch-like MSI-H ECs versus 44% in sporadic cases, driven by higher tumor mutational burden and CD68 + macrophage infiltration. While this study did not involve epidrugs, it suggests that epigenetic profiling could enhance patient selection for immunotherapy, a strategy epidrugs could augment by modulating tumor mutational burden or immune cell dynamics. However, the RO6870810 trial’s immunosuppressive effects, including reduced CD14 +/CD11b + monocytes, indicate that epidrugs may counteract immune activation if not carefully dosed or sequenced, necessitating refined approaches [231].

In conclusion, epidrugs offer significant potential in gynecological immunotherapy by altering epigenetic barriers to immune activation and sensitizing tumors to ICIs and chemotherapy. Clinical trials demonstrate modest efficacy but highlight challenges, including toxicity and context-dependent outcomes. Future efforts should focus on optimizing epidrug regimens, leveraging biomarkers for patient selection, and developing safer combinations to improve therapeutic outcomes in gynecological cancers.

## Conclusion and perspective

The integration of epigenetic regulation into immunotherapy strategies has ushered in a transformative era, redefining therapeutic approaches for these cancers. This review has comprehensively explored the critical role of epigenetic modifications encompassing DNA methylation, histone acetylation, histone methylation, RNA methylation, and histone lactylation in shaping the TIME and enhancing the efficacy of immunotherapies such as ICIs, cancer vaccines, cytokine-based therapies, and CAR T-cell therapies. By dynamically modulating immune cell infiltration, antigen presentation, immune checkpoint expression, and tumor immunogenicity, epigenetic mechanisms provide a robust framework for overcoming immunoresistance and improving clinical outcomes.

The evidence highlights the immunosuppressive consequences of aberrant epigenetic alterations, such as hypermethylation of immune-activating genes (e.g., IFN-γ, TAP1) and hypoacetylation driven by HDACs, which impair anti-tumor immunity in gynecological cancers. Conversely, hypomethylation of immunosuppressive genes like as VTCN1 and PD-L1 and acetylation-driven activation of immune checkpoints facilitate immune evasion. Preclinical and clinical studies demonstrate that epigenetic inhibitors, including DNMTi, HDACi, and BET inhibitors, can reprogram the TIME to enhance tumor immunogenicity. For example, DNMTi such as guadecitabine upregulate interferon pathways and neoantigen expression, sensitizing tumors to PD-1/PD-L1 blockade, while HDACi like vorinostat and belinostat restore MHC class II expression and promote CD8 + T-cell infiltration (Table 2 and 3). These agents, when combined with immunotherapies, exhibit synergistic effects, as evidenced by clinical trials reporting improved ORRs and PFS in ovarian and endometrial cancers.

However, significant Limitations And challenges hinder the seamless translation of epigenetic-immunotherapy combinations into routine clinical practice. First, clinical trials have revealed variable efficacy And toxicity profiles. For instance, the phase II study combining guadecitabine with pembrolizumab in platinum-resistant ovarian cancer reported a modest response rate of 6.6%, with only two partial responses, despite evidence of immune priming through LINE1 hypomethylation And increased cytotoxic T-cell activity. Similarly, the phase 1b study of the BET inhibitor RO6870810 with atezolizumab was terminated early due to severe immune-related adverse events, including systemic immune activation, And a lack of synergistic Antitumor effects, with a response rate of only 5.6%. These findings underscore the challenge of achieving optimal therapeutic synergy while managing toxicity, which may arise from non-specific epigenetic modulation or unintended immune suppression. Second, the context-dependent effects of epidrugs, influenced by tumor subtype, epigenetic profile, and immune landscape, complicate their application. For example, the efficacy of DNMTi varies between hypermethylated and hypomethylated ovarian cancer subtypes, and the immunosuppressive effects of BET inhibitors in some settings highlight the need for precise patient stratification. Third, suboptimal dosing and delivery remain critical barriers. The early termination of a trial combining decitabine with carboplatin due to high toxicity (60% grade 3/4 neutropenia) and reduced efficacy illustrates the difficulty in balancing epigenetic reprogramming with tolerable pharmacokinetics. Tumor penetration challenges further limit the ability of epidrugs to effectively target the TIME, particularly in solid tumors with dense stromal barriers. Fourth, the lack of robust biomarkers to predict response to epigenetic-immunotherapy combinations hampers personalized treatment. While MSI-H endometrial cancers and high tumor mutational burden are associated with better ICI responses, epigenetic biomarkers remain underexplored, and their integration into clinical decision-making is nascent. Finally, the complexity of the TIME, driven by interactions between epigenetic modifications, metabolic reprogramming (e.g., Warburg effect, lactate-driven histone lactylation), and immune cell dynamics, poses a challenge in designing combination therapies that comprehensively address all immunosuppressive mechanisms. These limitations highlight the need for rigorous optimization of epidrug regimens, improved delivery systems, and advanced biomarker discovery to maximize clinical impact.

Looking forward, the future of immunoepigenetic strategies in gynecological cancers lies in precision oncology. Advances in high-throughput sequencing, spatial transcriptomics, and multi-omics integration offer unprecedented opportunities to map epigenetic landscapes and their interactions with immune components, facilitating the identification of novel biomarkers and therapeutic targets. The development of next-generation epidrugs with enhanced specificity, such as selective HDAC6 inhibitors or EZH2 inhibitors, promises to minimize off-target effects and improve therapeutic indices. Furthermore, integrating epigenetic therapies with emerging immunotherapies, such as bispecific antibodies such as cadonilimab and dual-target CAR-T cells, could address tumor heterogeneity and antigen escape, enhancing response durability. Multi-omics approaches, combining epigenomic, transcriptomic, and proteomic data, will be crucial in elucidating the molecular underpinnings of immunotherapy resistance and tailoring combination regimens to individual patients. Additionally, innovative drug delivery systems, such as nanoparticle-based carriers, could improve tumor penetration and reduce systemic toxicity, addressing current limitations in epidrug administration.

In conclusion, the convergence of epigenetic regulation and immunotherapy represents a paradigm shift in the management of gynecological cancers. By targeting epigenetic mechanisms to reprogram the TIME, these strategies offer a promising avenue to overcome resistance, enhance anti-tumor immunity, and improve survival outcomes. Despite challenges in efficacy, toxicity, and biomarker development, continued investment in translational research, clinical trials, and precision medicine approaches will be essential to fully realize the potential of immunoepigenetic therapies. As the field advances, the integration of epigenetic interventions with immunotherapy holds the promise of transforming gynecological oncology, delivering personalized, effective, and durable treatments to patients facing these devastating diseases.

## Declaration of generative AI and AI-assisted technologies in the writing process

During the preparation of this work, the authors used ChatGPT by OpenAI to improve paper readability. After using this tool/service, the authors reviewed and edited the content as needed and took full responsibility for the publication's content.

## Data Availability

No datasets were generated or analysed during the current study.
